# Alx3 deficiency disrupts energy homeostasis, alters body composition, and impairs hypothalamic regulation of food intake

**DOI:** 10.1007/s00018-024-05384-z

**Published:** 2024-08-12

**Authors:** Mercedes Mirasierra, Antonio Fernández-Pérez, Blanca Lizarbe, Noelia Keiran, Laura Ruiz-Cañas, María José Casarejos, Sebastián Cerdán, Joan Vendrell, Sonia Fernández-Veledo, Mario Vallejo

**Affiliations:** 1 Instituto de Investigaciones Biomédicas Sols-Morreale, Consejo Superior de Investigaciones Científicas/Universidad Autónoma de Madrid, Madrid, Spain; 2 Centro de Investigación Biomédica en Red de Diabetes y Enfermedades Metabólicas Asociadas CIBERDEM, Instituto de Salud Carlos III, Madrid, Spain; 3 Department of Biochemistry, Universidad Autónoma de Madrid, Madrid, Spain; 4 Department of Endocrinology and Nutrition, Research Unit, Institut d’Investigació Sanitària Pere Virgili (IISPV) - Hospital Universitari de Tarragona Joan XXIII, Universitat Rovira i Virgili, Tarragona, Spain; 5 Chronic Diseases and Cancer Area 3, Instituto Ramón y Cajal de Investigaciones Sanitarias (IRYCIS), Madrid, Spain; 6 Neuropharmacology Laboratory, Neurobiology Department, Hospital Universitario Ramón y Cajal, Instituto Ramón y Cajal de Investigaciones Sanitarias (IRYCIS), Madrid, Spain; 7 Present Address: Centro para el Desarrollo Tecnológico e Industrial (CDTI), Madrid, Spain

**Keywords:** Alx3, Proopiomelanocortin, MC3R, Energy homeostasis, Body mass composition, Metabolic partitioning

## Abstract

The coordination of food intake, energy storage, and expenditure involves complex interactions between hypothalamic neurons and peripheral tissues including pancreatic islets, adipocytes, muscle, and liver. Previous research shows that deficiency of the transcription factor Alx3 alters pancreatic islet-dependent glucose homeostasis. In this study we carried out a comprehensive assessment of metabolic alterations in Alx3 deficiency. We report that Alx3-deficient mice exhibit decreased food intake without changes in body weight, along with reduced energy expenditure and altered respiratory exchange ratio. Magnetic resonance imaging reveals increased adiposity and decreased muscle mass, which was associated with markers of motor and sympathetic denervation. By contrast, Alx3-deficient mice on a high-fat diet show attenuated weight gain and improved insulin sensitivity, compared to control mice. Gene expression analysis demonstrates altered lipogenic and lipolytic gene profiles. In wild type mice Alx3 is expressed in hypothalamic arcuate nucleus neurons, but not in major peripheral metabolic organs. Functional diffusion-weighted magnetic resonance imaging reveals selective hypothalamic responses to fasting in the arcuate nucleus of Alx3-deficient mice. Additionally, altered expression of proopiomelanocortin and melanocortin-3 receptor mRNA in the hypothalamus suggests impaired regulation of feeding behavior. This study highlights the crucial role for Alx3 in governing food intake, energy homeostasis, and metabolic nutrient partitioning, thereby influencing body mass composition.

**Supplementary Information:**

The online version contains supplementary material available at 10.1007/s00018-024-05384-z.

## Introduction

The regulation of energy homeostasis requires the coordinated interaction of central and peripheral mechanisms to control food intake, fuel storage and distribution, and energy expenditure. Dysfunction of any of these mechanisms frequently results in two of the most common metabolic disorders, obesity, and diabetes, which in turn trigger the development of important complications.

In the periphery, pancreatic islets are key players in metabolic regulation due to their primary role in the maintenance of glucose homeostasis. Fluctuations in blood glucose concentrations are detected by β and α cells to secrete insulin or glucagon in response to increased or decreased glucose levels, respectively. In the first case, insulin promotes glucose uptake by target organs such as the liver, muscle, and adipose tissue, whereas in the second case, glucagon stimulates hepatic glucose release into the bloodstream.

Glucose-dependent hormone secretion in pancreatic islets requires the maintenance of complex cell-specific protein networks that are assembled during embryonic development. During this process, transcription factors acting coordinately in a hierarchically organized manner activate lineage-specific gene expression programs to define cell-specific functions. In previous studies, we identified the transcription factor Alx3 as a contributor to the regulation of insulin gene expression [1] and we found that, in turn, *Alx3* gene expression is under the control of Pdx1, a key β-cell-specific transcription factor essential for insulin gene expression and β-cell survival [2]. On the other hand, increased glucose levels result in stimulation of Alx3 synthesis [3], and in pancreatic α cells, this response has important consequences for the regulation of glucagon gene expression. Increased expression of Alx3 induced by glucose promotes interactions with the transcription factor PAX6, thus preventing its binding to the glucagon gene promoter and resulting in downregulation of glucagon gene expression [4].

The importance of Alx3 in the regulation of pancreatic islet function is underscored by the observation that *Alx3*-deficient mice exhibit mild hyperglycemia and glucose intolerance related to decreased insulin content, dysfunctional glucose-dependent insulin secretion, and increased β cell apoptosis, although these animals do not become overtly diabetic [5].

The contribution of Alx3 as a regulator of metabolic homeostasis has been associated to date with the pancreatic islet dysfunction observed in adult *Alx3*-deficient mice, and fittingly, no other readily detectable metabolic defects have been described in these animals. During development, *Alx3* has important roles in the formation of craniofacial structures and skin pigmentation patterns [3, 6–8]. Indeed, Alx3 deficiency in mice can lead to lethal neural tube closure defects, and in humans, mutations in the *ALX3* gene are associated with inborn facial midline defects and frontonasal dysplasia [7, 9]. However, the expression of *Alx3* in the head mesenchyme and neural crest cells associated with these phenotypes occurs transiently in the embryo, so adult mutant mice unaffected due to low penetrance of the *Alx3* inactivation are apparently normal except for the presence of mild hyperglycemia and glucose intolerance described above.

Here, we investigated in more detail the metabolic phenotype generated as a consequence of Alx3 deficiency using mice subjected to standard and hypercaloric dietary conditions. We report that adult *Alx3*-deficient mice exhibit reduced food intake, decreased energy expenditure, and altered body mass composition. As this phenotype is unrelated to pancreatic islet function, we tested the hypothesis that Alx3 could be expressed in hypothalamic nuclei regulating feeding and energy balance. We localize the expression of Alx3 in neurons of the arcuate nucleus and suggest new functions possibly related to the central regulation of metabolic homeostasis.

## Methods

### Mice

*Alx3* mutant mice [6] from a colony maintained in our institute on a hybrid FVBxC57BL/6J background [4] or backcrossed into a pure C57BL/6J background for more than 12 generations were used and genotyped as described [7]. FVBxC57BL/6J mice were used for all experiments except for those described in Supplementary Fig. 7, which were carried out with mice on a pure C57BL/6J background. All mice from either background were males of 16–20 weeks of age. Mice were housed under a 12:12-h dark/light cycle at 22 ± 2 °C. Experimental protocols were approved by institutional ethics committees (IIBM Ethics Committee on Human and Animal Experimentation and CSIC Ethics Committee) and comply with European Union (63/2010/EU) and Spanish (RD 53/2013) legislation.

### Fat mass

The relative amount of fat in each animal was calculated using magnetic resonance imaging (MRI) [10]. Mice were anaesthetized with 2% isoflurane and MRI was performed with a 7.0T 16 cm bore Bruker Biospect MRI system equipped with a quadrature 40 mm coil and a 90 mm gradient coil insert (maximum intensity 360 G/cm) (Bruker Medical Gmbh, Ettlingen, Germany). T2-weighted spin echo images were obtained using respiratory triggering and a rapid acquisition with relaxation enhancement (RARE) sequence. Axial and coronal images were taken from each animal. For axial planes, non-fat suppressed and fat suppressed images were acquired from each animal. For coronal planes, the fat signal was obtained by applying a frequency selective radiofrequency pulse with an irradiation offset of 1000 to 1100 Hz with respect to water to excite a spectral band of fat protons only. The acquisition parameters were: repetition time, 3200 ms; echo time 44 ms; number of averages 2; matrix, 256 × 256 within a field of view of 50 × 50 mm; number of slices, 14 for axial and 36 for coronal; slice thickness, 1.5 mm, without gap. Image analysis was carried out using NIH ImageJ. For each animal, the total fat volume was calculated and converted to grams by assuming a density of adipose tissue of 0.9 g/ml [11]. Fat mass was expressed as a percentage relative to the total weight of the animal.

### In vivo proton magnetic resonance spectroscopy (^1^H-MRS)

Non-localized ^1^H-MRS was performed using stimulated echo acquisition mode spectroscopic sequence without pulse water suppression, repetition time 1 second, 128 averages. Spectroscopic data were acquired using 2018 points and 7.5 kHz spectral width. The intensities of the water and fat signals were calculated using the Mnova software (Mestrelab Research, Santiago de Compostela, Spain).

### Blood glucose determinations

Glucose in blood obtained by tail puncture was measured using a glucometer. For glucose tolerance tests (GTT), determinations were performed at baseline after fasting overnight and 15, 60 and 120 min after the injection of glucose (2 g/kg, i.p.). For insulin tolerance tests (ITT), glucose concentrations were determined at baseline after a 4-hour fast and then at the indicated times after the injection of insulin (Actrapid; Novo Nordisk, Bagsvaert, Denmark; 0.75 U/kg i.p.).

### Noradrenaline content

Mice were decapitated and epididymal visceral white adipose tissue (vWAT) and the gastrocnemius muscles were rapidly removed and frozen in liquid nitrogen. The muscles were homogenized in 0.4 N perchloric acid containing 0.2 mM EDTA and 0.5 mM Na_2_S_2_O_5_, and centrifuged at 10,000 g at 4 °C for 20 min. Noradrenaline levels were determined from the resulting supernatant by HPLC using an ESA Coulochem detector [12].

### Reverse transcription-quantitative PCR (RT-qPCR)

Total RNA was extracted using Trizol (Thermo Fisher Scientific, Madrid, Spain) from the following tissues: a dissected hypothalamic block containing the arcuate nucleus, a fragment of the spinal cord corresponding to the lumbar and part of the thoracic levels, one gastrocnemius muscle, brown adipose tissue (BAT), liver or vWAT. The dissection of the hypothalamus was performed as described [13]. Briefly, the brain was placed on ice with the ventral surface facing up and coronal cuts made at the level of the caudal edge of the optic chiasm and the rostral edge of the mammillary bodies. From the resulting slice, the ventral hypothalamus was separated by two parasagittal cuts located 1 mm at either side of the midline, and a horizontal cut located at half the dorso-ventral length of the third ventricle. The arcuate nucleus was separated from this block within a piece of tissue generated by converging bilateral 45º cuts with lower ends separated 1 mm on the median eminence. RT-qPCR was performed in the Core Facilities of the Genomics Unit at the Scientific Park of Madrid (http://fpcm.es). TaqMan primers (Applied Biosystems, Alcobendas, Madrid, Spain) or SYBR Green detection (Applied Biosystems) with specific primers indicated in Supplementary Table 1 were used. Reactions were performed in triplicate and values were normalized to β2 microglobulin (hypothalamus), *Hprt* (liver, adipose tissue and muscle) or *Gapdh* (spinal cord) mRNA levels [13, 14] using the double delta Ct method [15].

### α-MSH content

Arcuate nuclei dissected as described above were immediately frozen in dry ice. A mild RIPA buffer (50 mM Tris-HCl, pH 7.5; 150 mM NaCl; 1 mM EDTA; 1% sodium deoxycholate; 0.1% SDS) was used to homogenize and then sonicate the samples. After centrifugation at 14,000 g at 4 °C for 5 min, the supernatants were used for determination of α-MSH levels using a mouse αMSH ELISA Kit (ref. EM1454; Wuhan Fine Biotech Co., Wuhan, China) following the instructions provided by the manufacturer. The intra- and inter-assay coefficients of variation are 4.82% and 4.8%, respectively, for samples in the range of ~ 39 pg/ml. The assay standard curve was prepared with concentrations of αMSH ranging from 4.69 to 300 pg/ml, and our lineal range was between 10 and 150 pg/ml. The kit is based on double antibody-sandwich detection method specific for αMSH with no obvious cross reaction with other analogues.

### Indirect calorimetry

Indirect calorimetry was performed as described [13] using a PhenoMaster monitoring system (TSE Systems GmbH, Bad Homburg, Germany). Oxygen consumption and CO_2_ production were directly measured over a period of 48 to 72 h, and from these values respiratory exchange ratio (RER) and energy expenditure were calculated. Locomotor activity was monitored by counting infrared photocell beam breaks on the X and Y axes.

### Food intake

Food intake was automatically monitored for four consecutive days while the mice were in the metabolic chambers using specific weight sensors connected to the hanging food basket in each cage. For re-feeding experiments, singly housed mice were acclimatized with food provided *ad libitum* for one week. Food was then withdrawn at 20:00 h and mice were fasted until 08:00 h. At this time food was provided *ad libitum* and intake was measured every 15 min for a period of 5 h, after which the arcuate nucleus was removed for determination of *Pomc* and cocaine- and amphetamine-regulated transcript (*Cart*) mRNA expression by RT-qPCR.

To test the effects of D-Trp8-γMSH [16], mice were acclimatized for three days prior to the beginning of the experiments, and then they were injected with saline (i.p.) for two consecutive days. On the following day, either saline or D-Trp8-γMSH (0.2 mg/kg i.p.) (Santa Cruz Biotechnology) were injected. Treatments took place 30 min before lights were off, and food intake was determined 3, 6 and 12 h later.

### Muscle volume

We performed MRI as described [17] using the equipment described above. T2-weighted spin echo images were acquired with a rapid acquisition with relaxation enhancement sequence in axial and coronal orientations with the following parameters: TR = 3000 ms, TE = 11.57 ms, RARE factor = 8, Av = 12, FOV = 2.5 cm, acquisition matrix = 256 × 256 corresponding to an in plane resolution of 97 × 97 µm^2^, slice thickness = 1 mm without gap between slices. Hind limb images were analyzed with NIH ImageJ software on five consecutive slices and values from both limbs were added to generate a single value for each mouse. For coronal planes, the first slice was located in the dorsal direction following the one showing the entire length of the tibia. For axial planes, the stack started at the third slice distal to the tibial plateau. In each slice, regions of interest were manually traced in each projection, and areas of hyper- or hypointense signal corresponding to fat or bone, respectively, were excluded.

### Rota-rod

Motor performance on an accelerating rota-rod apparatus (Hugo Basile, Italy) was tested as described [12]. Rotation accelerated from 4 to 40 rpm within 5 min and the time to fall was measured. Inter trial interval was 15 min.

### Muscle strength test

Fore limb and all limb strength tests were performed using a digital grip dynamometer (PCE-FM 50 N, PCE Instruments, Torralba, Albacete, Spain) with a range of 0 to 50 N and a resolution of 0.01 N as described [18]. Values (in grams force) were recorded and the average of three trials spaced at 2 min intervals was used for analysis.

### Treadmill

Motor performance during acute exercise was monitored in a closed treadmill chamber (TSE Systems GmbH, Bad Homburg, Germany) tilted at an angle of 12.5 ° [19]. The treadmill speed was initially adjusted to 0.15 m/s for 2 min, and then increased at 0.2 m/s for another two minutes. After this, an acceleration protocol was introduced at a rate of 0.05 m/s every two minutes. An electrode located at the lower end of the treadmill delivered a small electric discharge (1 mA) to encourage mice to run. The elapsed time to the first shock was recorded and scored as the latency to fatigue. A cut-off time of 21 min equivalent to a speed of 0.65 m/s was established. During exercise, oxygen consumption was monitored at one minute intervals using a TSE PhenoMaster monitoring system.

### Muscle histology

Paraffin-embedded tissue sections  (5 μm) of the gastrocnemius muscle were cut and stained with hematoxylin and eosin. Myofiber cross-sectional area was determined with Image J software from digital images counting approximately 200 fibers per muscle section.

### Western blots

Protein extracts were prepared from a small block of ventral hypothalamic tissue containing the arcuate nucleus freshly dissected on ice as described [13]. As positive controls for expression of Alx3, we used protein extracts from primary mouse embryonic mesenchyme (pMEM), MIN6, or transfected COS-7 cells [1, 3, 20], or from mouse embryo forehead mesenchyme obtained at 10.5 days of gestation [20]. For the preparation of muscle lysates, the gastrocnemius muscles were readily dissected and immediately frozen in liquid nitrogen. Samples were homogenized in 200 µl of lysis buffer containing freshly added protease inhibitors as indicated [21]. After centrifugation (14,000 rpm, 5 min at 4 °C), the supernatant was collected and stored at − 20° C. Immunoreactivity was detected with commercial antibodies indicated in Supplementary Table 2. For Alx3, we used a specific antibody directed against the N-terminal region (1:4000 dilution) [20]. Bands were visualized with ECL (GE Healthcare, USA) and densitometry was performed using ImageJ (http://imagej.net).

### Immunohistochemistry and immunofluorescence

Brains were fixed by transcardial perfusion with 4% paraformaldehyde, removed and cryoprotected in PBS containing 20% sucrose. After antigen retrieval (25 min at 80 ºC in 10 mM citrate buffer, pH 8.5), free-floating coronal cryostat sections (30 μm) were incubated for 72 h at 4 ºC with the Alx3-specific antibody (1:2000 dilution) [20]. Pancreas immunohistochemistry was performed as described [1]. For pre-absorption experiments, the Alx3 antibody was previously incubated overnight at 4ºC with control GST or GST-Alx3ΔC fusion protein (500 ng/ml) [20]. Immunodetection was carried out with a secondary biotinylated goat antirabbit antiserum using nickel-intensified immunoperoxidase staining. For immunofluorescence in the arcuate nucleus, we used brains from POMC-GFP mice (C57BL/6J-Tg(Pomc-EGFP)1Low/J) [16] and AgRP^tdTomato^ mice [22]. Coexpression of Alx3 with either POMC- or AgRP-neurons was assessed using coronal sections (20 μm) spanning the entire arcuate nucleus (bregma − 1.1 to -2.6 mm), after incubation with the Alx3 antibody and secondary Alexa Fluor-555 or Alexa Fluor-488 rabbit antibodies (Invitrogen). Sections from three different mice were visualized on a confocal LSM710 microscope (Zeiss). For brainstem immunofluorescence, coronal sections (30 μm) were incubated with antibodies against Alx3 and tyrosine hydroxylase followed by Alexa Fluor-546 or Alexa Fluor-647 mouse antibodies (Invitrogen). For developmental studies, embryonic day 13.5 (E13.5) mouse embryos were fixed in phosphate-buffered 4% paraformaldehyde and cryoprotected. Sagittal cryostat sections (8 μm) were processed for immunofluorescence using β-galactosidase and adrenocorticotropin hormone (ACTH) antibodies followed by Alexa Fluor-488 and Alexa Fluor-546. Brainstem and embryo sections were visualized using a Stellaris 8 TauSTED super-resolution confocal microscope (Leica). Specifications of the antibodies used are indicated in Supplementary Table 2.

### **Positron emission tomography and computerized tomography (PET-CT) imaging**

Mice were fasted overnight and then blood glucose levels were measured from the tail vein using a glucometer (Glucotrend Soft Test System, Boehringer Ingelheim, Mannheim, Germany). Each mouse received an injection of the radiotracer (11.1 MBq) 2-deoxy-2-(^18^F)fluoro-D-glucose (^18^F-FDG, 300 µCi in 0.2 ml i.p.). The animals were anaesthetized 45 min later with 2% isofluorane for image acquisition. A PET-CT Albira ARS hybrid tomograph (Oncovision, Valencia, Spain) with a PET resolution of 1.5 mm (axial and transaxial fields of view, 4 and 8 cm, respectively) was used. PET acquisition was performed during 20 min and was followed by a CT scan. Image reconstruction was carried out using an ordered subset expectation maximization (OSEM) iterative algorithm (3 iterations), and data were corrected for random events, scatter and decay.

To evaluate ^18^F-FDG uptake in different brain regions, image analysis was carried out using the PMOD 3.0 suite (Pmod Technologies, Zurich, Switzerland). Volumes of interest (VOI) were defined on a magnetic resonance image template [23] that was made to coincide with the PET-CT co-registered images and the tracer activity (kBq/ml) was determined. For quantification, the standardized uptake value (SUV) was calculated as the ratio between radioactivity concentration (kBq/ml) and the injected dose (kBq) divided by body weight. Values were normalized to whole brain.

### Functional diffusion weighted magnetic resonance imaging (fDWI)

Mice fed *ad libitum* of after a 16 h overnight fast were anaesthetized with 2% isofluorane. For fDWI a 7.0T Bruker Biospect scanner equipped with a 23 mm mouse head resonator within a 90 mm gradient coil (36G/cm) was used as described [24], and images were acquired with a Linux-based console using Paravision 5.1 software (Bruker BioSpin MRI GmBH, Ettlingen, Germany). Briefly, serial consecutive images of an axial slice containing the hypothalamus were obtained using nine “high b” (300 < b < 2000 s/µm^2^) values in three orthogonal directions: Head-Foot (H-F), Left-Right (L-R) and Antero-Posterior (A-P). Acquisition parameters were: Repetition time, 3200 ms; echo time, 31 ms; number of averages, 4; duration of the diffusion gradients, δ = 4 ms; and separation between the diffusion gradients, ∆=20 ms.

Hypothalamic arcuate, ventromedial and dorsomedial nuclei were identified by superimposing the magnetic resonance images to those of a mouse brain atlas [25], and regions of interest corresponding to each nucleus were selected manually. Images were analyzed using a monoexponential model of attenuation of the DWI signal. Parameters were determined with the non-linear least-square fitting Trust-Region algorithm, restricting the goodness of fit to be higher than 0.8 for optimization [26].

### Leptin sensitivity tests

Mice were acclimatized by subjecting them to handling and sham injections, and on the day previous to the test they were fasted overnight. After this, vehicle (0.9% NaCl) or recombinant mouse leptin (3 µg/g) (Peprotech catalogue nº 450−31) was injected i.p. One or two hours after the injection samples from the arcuate nucleus were prepared for determination of STAT3 phosphorylation by western blot or SOCS3 expression by RT-qPCR, respectively.

### Statistics

Data are expressed as mean ± s.e.m. Unless otherwise indicated, data were analyzed by two-way ANOVA followed by Bonferroni transformation or by two-tailed unpaired Student’s *t*-test as appropriate, using Package GraphPad Prism 9 software.

Indirect calorimetry data for body mass-dependent variables were further analyzed by ANCOVA/GLM (Generalized Linear Model) as described [27] using CalR (https://calrapp.org/) to test for possible interactions between mass and genotype, whereas mass independent variables such as RER and locomotor activity were tested by ANOVA.

Apparent diffusion coefficient (ADC) data obtained in fDWI experiments were analyzed using R software (https://www.r-project.org/) by an ad hoc-designed fitting to linear mixed-effects (LME) models as previously described in detail [28]. Briefly, ADC values were fitted to several LME regression models using the *lme* function of *nlme* package and following a multilevel selection strategy on linear combinations of each genetic condition (WT or KO), feeding status (fed or fasted), hypothalamic nucleus (arcuate, ventromedial or dorsomedial), and the corresponding double (genetic condition: feeding status, genetic condition: nuclei or feeding status: hypothalamic nuclei) and triple interactions, as fixed effects or predictor variables. The nested experimental design with intra-animal measures of the three hypothalamic nuclei in two feeding conditions was modelled by adding a random term to the model “animal over nuclei over feeding status”. On the best fitting model, type III ANOVA tests were performed with the *Anova* function of the *car* package, and differences between feeding conditions were assessed for each genotype and hypothalamic nucleus using the *emmeans* package.

## Results

### *Alx3*-deficient mice show contrasting responses to dietary regimens on body composition and energy balance

**Fig. 1:**
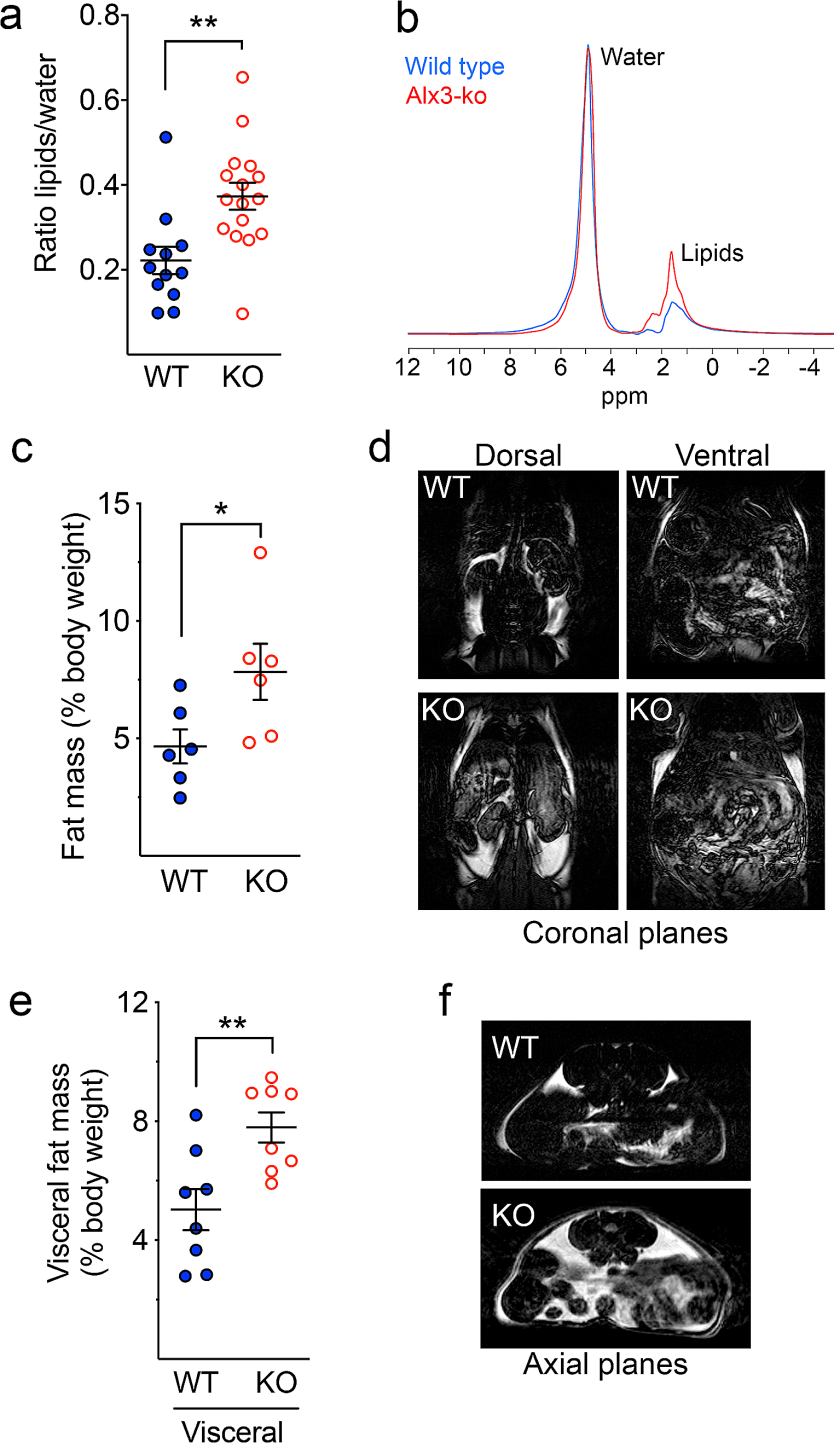
Alx3-deficient mice show increased proportion of fat mass **A**) Quantification of the total proportion of body lipids by ^1^H-MRS (*n* = 12 wild type and 16 Alx3-deficient mice). **B**) Representative spectra from which measurements were carried out. **C**) Total fat mass relative to body weight calculated from coronal MRI planes (*n* = 6 mice per genotype). **D**) Representative MRI coronal planes used for quantification. The distance between the ventral and dorsal planes shown was 7.5 mm. **E**) Visceral fat mass relative to body weight calculated from axial MRI planes (*n* = 8 mice per genotype). **F**) Representative MRI axial planes used for quantification corresponding to the abdominal region. *p < 0.05; **p < 0.01, Student’s *t*-test

Our initial observations indicated that although *Alx3*-deficient mice exhibit body weight curves that are similar to those of wild type animals, the amount of food that they eat is significantly reduced relative to control mice (Supplementary Fig. 1). This apparent discrepancy suggested that Alx3 may be important for the regulation of fuel storage or utilization and energy homeostasis so that lower food intake and similar body weight in *Alx3*-deficient mice could reflect altered body composition. Indeed, ^1^H-MRS showed a higher proportion of total lipids associated with Alx3 deficiency (Fig. 1A and B). To specifically address this question, we performed MRI on coronal planes and confirmed that the percentage of total fat mass relative to body weight was higher in *Alx3*-deficient mice (Fig. 1C and D). Analyses of images on axial planes showed that the proportional amount of visceral adipose tissue relative to body weight was higher in *Alx3*-deficient mice (Fig. 1E and F). Confirming these results, we determined the absolute weight of visceral fat depots and found that it was significantly increased in *Alx3*-deficient mice as compared with wild type animals (2.10 *±* 0.13 versus 1.35 *±* 0.17 g, respectively; *P* < 0.01, Student’s *t*-test; *n* = 8 per group).

**Fig. 2:**
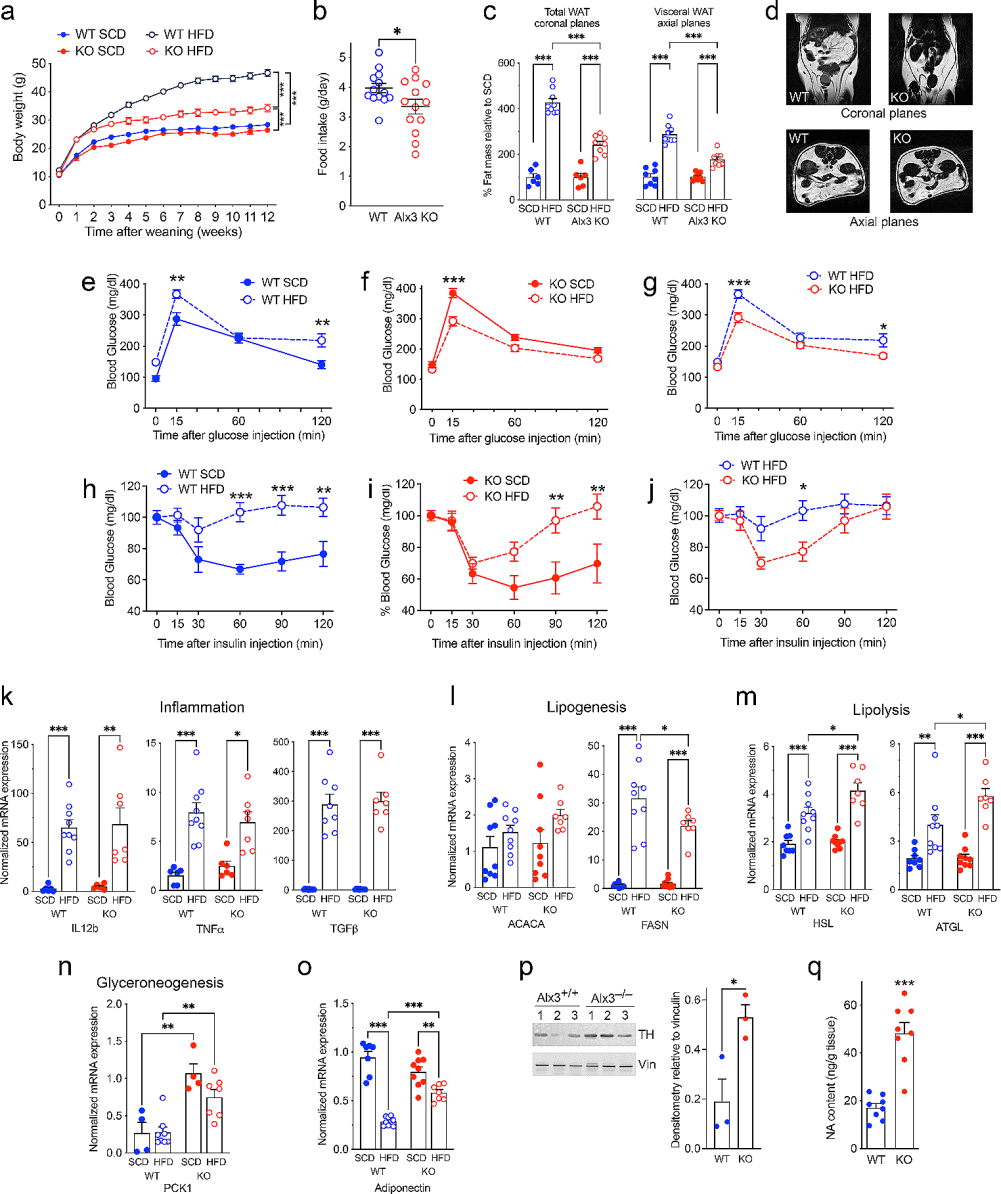
Altered response of *Alx3*-deficient mice to feeding with a high fat diet **A**) Body weight curves of *Alx3*-deficient and wild type mice fed with standard chow (SCD) or high-fat (HFD) diets (8–10 animals per group). **B**) Daily high-fat food intake determined at an age of 12 weeks. Values represent the mean of the total amount of grams per day taken by each mouse during a four-day period (*n* = 13 per group). **C**) Percentage of fat mass calculated from coronal or axial MRI planes relative to the amount of fat present in mice fed with SCD in each genotype (*n* = 6–9 mice per group). **D**) Representative MRI coronal planes used for quantification. **E**-**J**) Blood glucose levels observed during glucose tolerance tests performed after an overnight fast (**E**-**G**) or during insulin tolerance tests performed after a 4-hour fast (**H**-**J**) (*n* = 6 per group). In G and J the same results represented in the previous graphs have been plotted to facilitate comparisons. **K**-**O**) Expression of mRNAs from visceral white adipose tissue corresponding to genes encoding inflammation markers (**K**), lipid metabolism enzymes (**L**-**N**) and adiponectin (**O**) (*n* = 6–11 group). **P**) Left panel, western blots showing levels of tyrosine hydroxylase (TH) and vinculin (Vin) as a loading control. The numbers on top of each lane indicate individual mice from which samples were obtained. Right panel, densitometric quantification of the intensities of the bands. **Q**) Noradrenaline (NA) content in vWAT determined by HPLC (*n* = 8 per genotype). Data represent the mean ± SEM. *p < 0.05, **p < 0.01 and ***p < 0.001, two-way ANOVA (**A**, **C** and **E-N**) or Student’s *t*-test (**B**, **P**-**Q**)

To determine whether these differences were maintained in animals subjected to overnutrition, we placed mice after weaning on a high-fat diet (HFD, 60% fat content) for 12 weeks and compared the results with animals fed a standard chow diet (SCD). Surprisingly, body weight gain was significantly lower in *Alx3*-deficient than in control mice (Fig. 2A), consistent with decreased food intake under the HFD regime (Fig. 2B). MRI confirmed that the accumulation of fat mass induced by HFD was higher in wild type mice than in *Alx3*-deficient animals in proportional terms relative to the fat mass present in the SCD condition in either genotype (Fig. 2C and D). Importantly, the absolute weight of visceral adipose depots accumulated after the HFD regime was similar in both genotypes (wild type, 3.38 *±* 0.22 g; *Alx3*-ko 3.51 *±* 0.16 g).

Taken together, these results indicate that animals of both genotypes accumulate fat until they reach a similar maximum when exposed to HFD even though the *Alx3*-deficient mice store more fat than their wild type counterparts under a SCD regime. Thus, the proportional increment of fat accumulation from a SCD to a HFD condition is smaller in Alx3-deficicient mice, as indicated in Fig. 2C.

Since *Alx3*-deficient mice fed with a SCD are known to exhibit hyperglycemia and glucose intolerance [5], we assessed whether disparities in adiposity relative to control animals differentially affect insulin sensitivity. As expected, GTT and ITT showed that wild type mice fed with a HFD developed glucose intolerance and insulin resistance (Fig. 2E and H). On the contrary, glucose intolerance in *Alx3*-deficient mice reversed when animals were fed with HFD (Fig. 2F), showing blood glucose levels similar to those observed in SCD-fed wild type mice (compare Fig. 2F with 2E) and significantly lower than those observed in wild type mice fed on a HFD (Fig. 2G). In parallel, insulin resistance, that was evident in HFD-fed wild type mice relative to SCD-fed controls, was less pronounced in HFD-fed *Alx3*-deficient mice, which were more sensitive to insulin than controls (Fig. 2I and J). These data are in consonance with the observed increase in genes encoding the glucose transporter Glut2 and the glycogenic enzymes Pck1 and Gys1 in the liver of HFD-fed *Alx3*-deficient mice relative to HFD-fed wild type controls (Supplementary Fig. 2).

The expression of the proinflammatory genes Il-12b, TNFα and TGFβ was similarly increased in the vWAT of *Alx3*-deficient and control animals fed with HFD (Fig. 2K). In this tissue, the HFD-induced expression of the lipogenic gene *Fasn* was less pronounced in *Alx3*-deficient than in wild type mice (Fig. 2L), whereas the HFD-induced expression of the lipolytic genes *Hsl* and *Atgl* was relatively higher in mutant mice (Fig. 2M). No differences in the expression of lipolytic or lipogenic genes were found between *Alx3*-deficient and control mice fed with SCD. Interestingly, in *Alx3*-deficient mice, the expression of the glyceroneogenic gene *Pck1* [29] was higher than in wild type mice regardless of whether the animals had been fed with standard chow or with HFD (Fig. 2N). In addition, the expected decrease in adiponectin mRNA expression in vWAT induced by HFD was less pronounced in *Alx3*-deficient mice than in controls (Fig. 2O). Consistent with the predominant expression of lipolytic relative to lipogenic genes, suggesting increased sympathetic tone [30], the levels of the catecholamine synthesizing enzyme tyrosine hydroxylase, as well as noradrenaline content in vWAT, were higher in *Alx3*-deficient mice (Fig. 2P and Q).

**Fig. 3:**
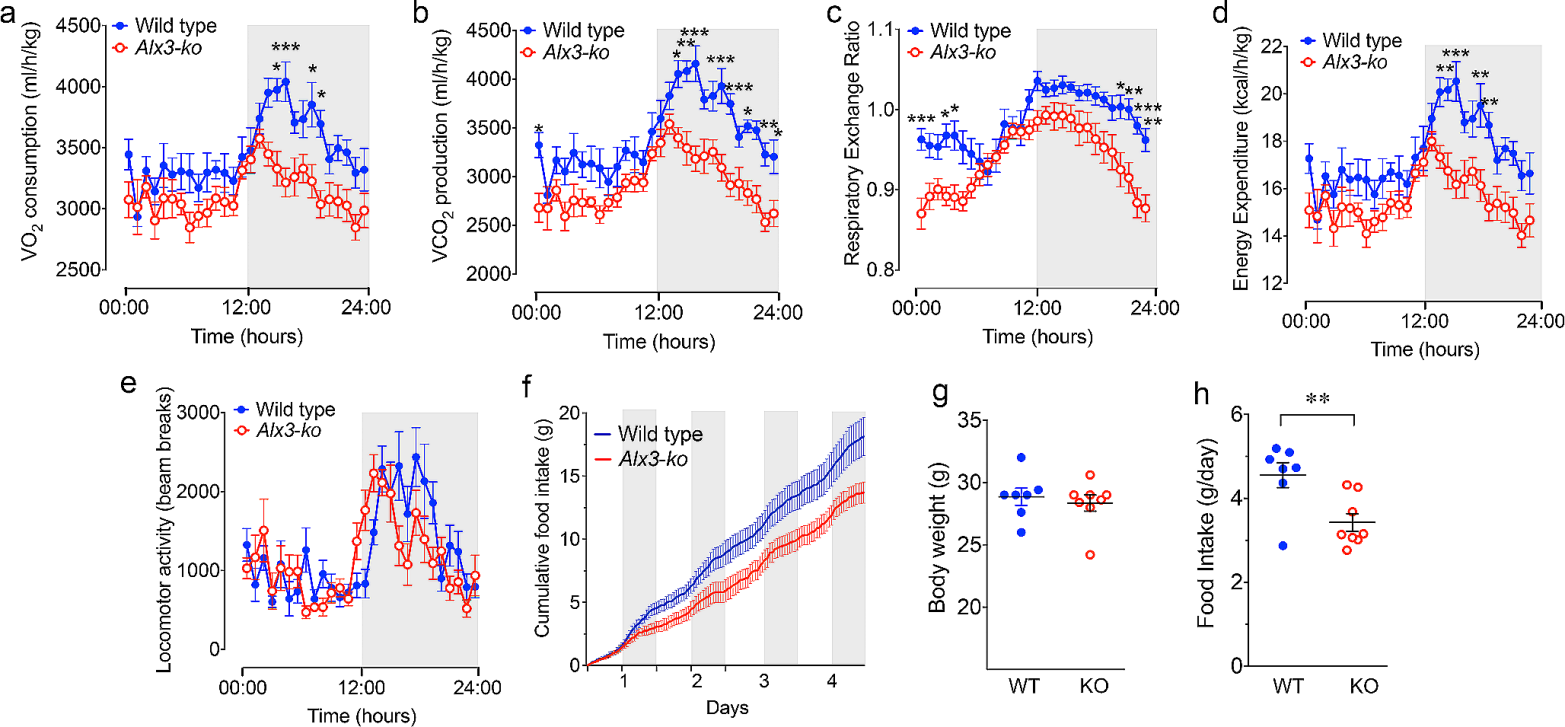
*Alx3*-deficient mice show altered energy expenditure and food intake, but not body weight **A**-**E**) Indirect calorimetry measurements showing O_2_ consumption, CO_2_ production, RER, energy expenditure and locomotor activity values corresponding to a complete 24-hour cycle. The data represent the mean values obtained at each time point over a period of three days for each mouse. Extended data showing the values corresponding to the entire three-day period are shown in Supplementary Fig. 3. **F**) Cumulative quantification of the amount of food eaten during four consecutive days. The dark phase of the 24-h cycle is represented in gray. **G** and **H**) Body weight and daily food intake (four days). Data represent mean *±* SEM. *p < 0.05, **p < 0.01, ***p < 0.001, two-way ANOVA followed by Bonferroni transformation (**A**-**E**) or Student’s *t*-test (**G** and **H**) (*n* = 7 wild type and 8 *Alx3*-ko mice)

Based on these data, we investigated whether Alx3 deficiency affects energy metabolism by performing indirect calorimetry experiments. We found that *Alx3*-deficient mice exhibited decreased O_2_ consumption, CO_2_ production, RER, and energy expenditure relative to control animals (Fig. 3A-D, Supplementary Fig. 3A-D and Supplementary Table 3). These differences were more clearly observed during the night, coinciding with the period of the 24-hour cycle in which the animals are more active. However, no differences were observed in locomotor activity between *Alx3*-mutant and wild type animals (Fig. 3E, Supplementary Fig. 3E and Supplementary Table 3). During these trials, reduced food intake but similar body weight were confirmed (Fig. 3F-H). In HFD-fed mice, oscillating O_2_ consumption, CO_2_ production, energy expenditure and locomotor activity were lower in *Alx3*-deficient than in wild type mice. RER values did not display significant diurnal rhythms as expected in mice subjected to a HFD regime [31], but also remained lower in *Alx3*-mutant mice (Supplementary Figs. 4 and 5 and Supplementary Table 4).

### *Alx3*-deficient mice show muscle atrophy and motor dysfunction with impaired neurotransmission

**Fig. 4:**
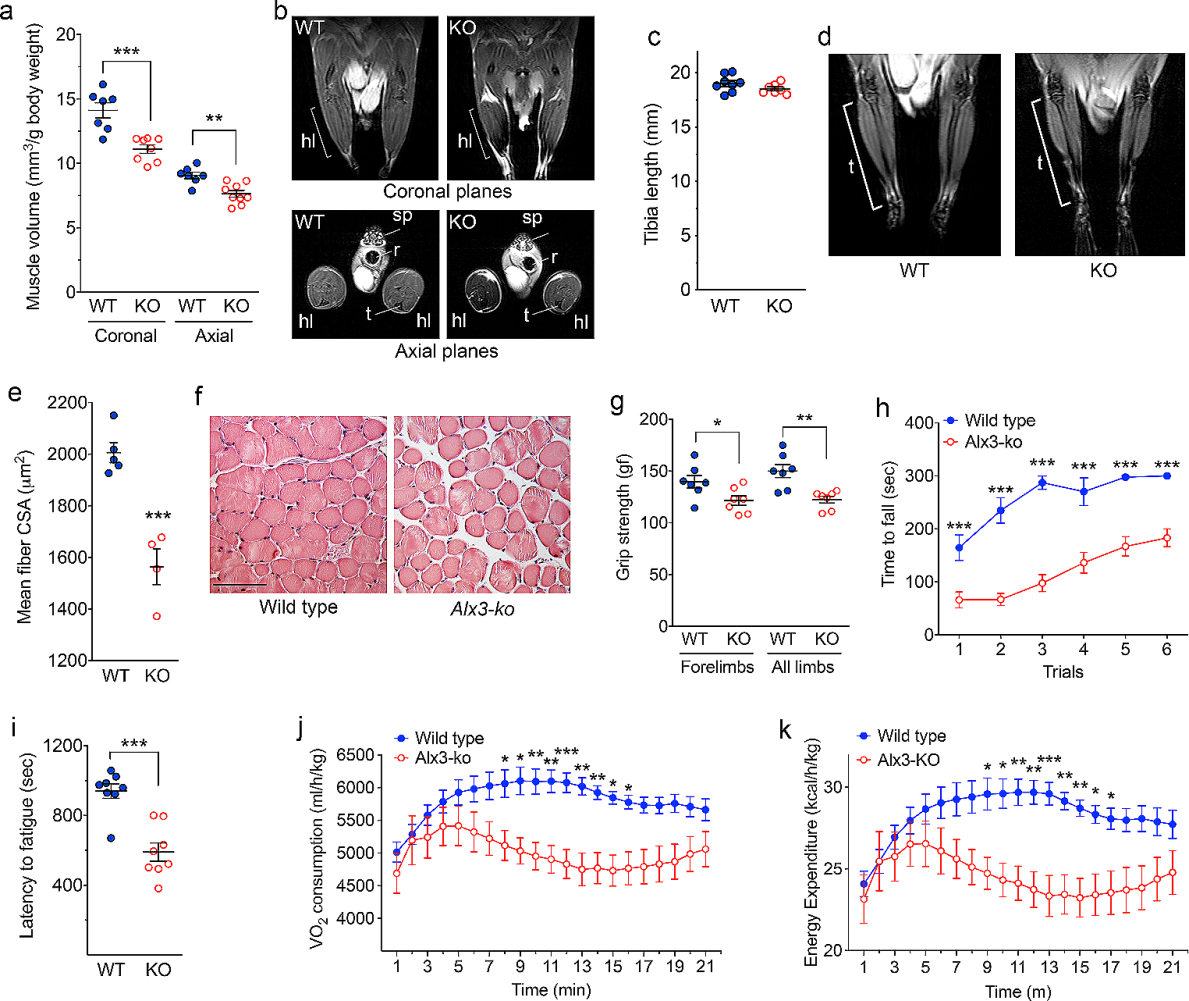
Decreased muscle volume and presence of motor dysfunction in *Alx3*-deficient mice **A**) Muscle volume relative to body weight calculated from coronal or axial images generated by MRI from hind limbs of wild type or *Alx3*-ko mice (*n* = 7–9 mice per genotype). **B**) Representative MRI coronal and axial planes used for quantification. hl, hind limb; t, tibia; r, rectum; sp, spine. The brackets indicate the muscle region analyzed, corresponding to the length of the tibia. **C**) Tibia length measured from coronal images generated by MRI from wild type (*n* = 8) or Alx3-ko (*n* = 7) mice. **D**) Representative MRI coronal planes used for tibia length measurements. The brackets indicate the region analyzed corresponding to the length of the tibia. **E**) Mean myofiber cross-sectional area (CSA) determined in gastrocnemius muscle from wild type or *Alx3*-deficient mice stained with hematoxylin and eosin (*n* = 5 wild type and 4 *Alx3*-deficient mice). **F**) Representative images of transverse sections of gastrocnemius muscles from which CSA was determined. Scale bar = 100 μm. **G**) Muscle strength as assessed by the grip test (*n* = 7 mice per genotype). **H**) Decreased motor performance of *Alx3*-ko mice relative to wild type animals as assessed by the rotarod test (*n* = 8 wild type and 7 Alx3-deficient mice). **I**) Decreased latency to fatigue in Alx3-deficient mice during acute enduring exercise on a treadmill (*n* = 8 per genotype). **J** and **K**) Oxygen consumption and energy expenditure during acute exercise on the treadmill (*n* = 8 per genotype). *p < 0.05; **p < 0.01; ***p < 0.001, Student’s t test (**A**, **E**, **G** and **I**) or two-way ANOVA followed by Bonferroni transformation (**H**, **J** and **K**)

The discrepancies between fat mass and body weight observed in *Alx3*-deficient mice fed with standard chow and their unusual response to HFD led us to hypothesize a proportional reduction in lean mass in these animals. To test this, we performed MRI and found that muscle volume in hind limbs was reduced in *Alx3*-deficient mice relative to wild type mice (Fig. 4A and B). This reduction in muscle mass was not due to decreased body length, as indicated by the absence of significant differences in bone length measured on the tibia from magnetic resonance images (Fig. 4C and D). Consistently, histological analysis of the gastrocnemius muscle revealed decreased mean fiber cross-sectional area (Fig. 4E and F). This was accompanied by reduced grip strength relative to control animals (Fig. 4G). When tested for motor performance, *Alx3*-deficient mice scored significantly lower than wild type controls on the rotarod motor coordination test (Fig. 4H) and showed decreased time to fatigue (Fig. 4I) and reduced oxygen consumption and energy expenditure on the treadmill acute enduring exercise test (Fig. 4J and K).

We did not find significant differences in the expression of genes regulating glucose transport and metabolism or mitochondrial function in the gastrocnemius muscle (Supplementary Fig. 6), indicating that an intrinsic metabolic dysfunction is unlikely to be responsible for the muscle phenotype in *Alx3*-deficient animals. Importantly, the availability of *Alx3*-deficient mice on a pure C57BL/6J background allowed us to determine that these mice do not exhibit hyperglycemia or glucose intolerance, but retain increased adiposity and decreased muscle mass and strength (Supplementary Fig. 7), indicating that the muscle phenotype is unrelated to dysfunctional glucose homeostasis.

**Fig. 5:**
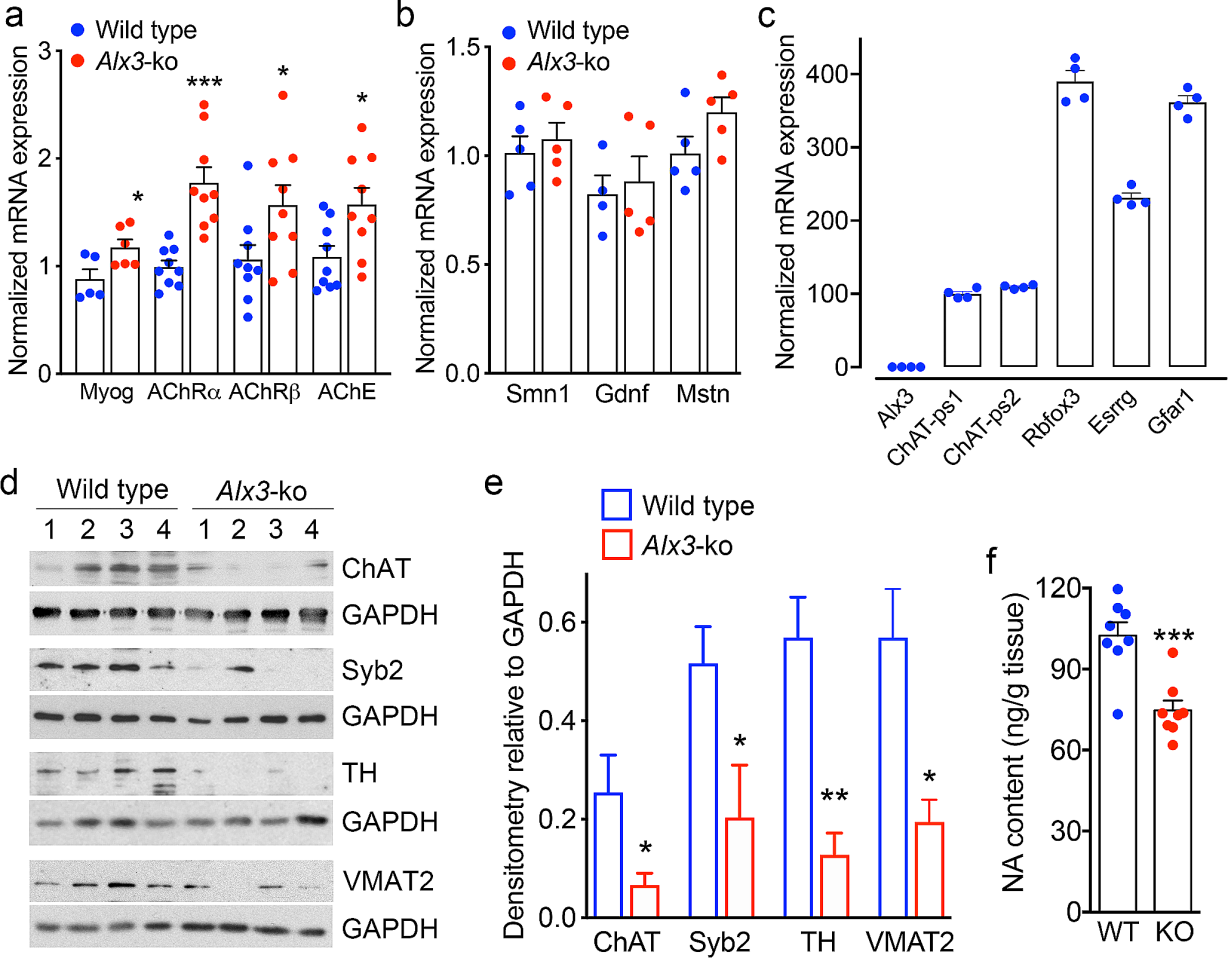
Altered neurotransmission and decreased noradrenergic input in muscles of *Alx3*-deficient mice (**A**) Expression of mRNAs from gastrocnemius muscle encoding the denervation markers myogenin (Myog), α and β subunits of the acetyl choline receptors (AChRα and AChRβ) and acetylcholinesterase (AChE) (*n* = 9 per genotype except for Myog, *n* = 6). (**B**) Expression of mRNAs from gastrocnemius muscle encoding the endogenous trophic factors survival of motor neuron-1 (Smn1), glial cell-derived neurotrophic factor (Gdnf) and myostatin (Mstn) (*n* = 5 per genotype). (**C**) Lack of expression of *Alx3* in the spinal cord of wild type mice. Expression of choline acetyltransferase (ChAT) detected with two different primer sets (ps1 and ps2) are shown as positive controls. Rbfox3, Esrrg and Gfar1 are selective markers of motor neurons (*n* = 4). (**D**) Western blots with lysates from the gastrocnemius muscle showing levels of choline acetyltransferase (ChAT), synaptobrevin-2 (Syb2), tyrosine hydroxylase (TH) and vesicular monoamine transporter 2 (VMAT2). The numbers on top of each lane indicate individual mice from which samples were obtained. (**E**) Densitometric quantification of the intensities of the bands from panel D. (**F**) Levels of noradrenaline in gastrocnemius homogenates from wild type (WT) and *Alx3*-ko mice (*n* = 8 per genotype). *p < 0.05; **p < 0.01, ***p < 0.001; Student’s *t*-test

Since reduced muscle mass can be associated with denervation or decreased neural input on the neuromuscular junction, we tested for the expression of genes known to be upregulated in response to denervation [32–34]. We found that in the gastrocnemius muscle, the expression of myogenin, the acetylcholine receptor (AChR) subunits α and β, and acetylcholinesterase was higher in *Alx3*-deficient that in control mice (Fig. 5A). In contrast, expression of *Gdnf* [35, 36], myostatin [37] and *Smn1* [38] was similar in both genotypes (Fig. 5B), suggesting that changes of neuromuscular junction proteins were not myogenic in origin. To test whether these changes were possibly due to a direct effect of Alx3 deficiency in motor neurons, we tested for expression of Alx3 in the spinal cord of wild type mice using the acetyl choline-synthetizing enzyme choline acetyltransferase (ChAT) and the selective motor neuron markers Rbfox3, Esrrg and Gfar1 [39] as controls, but we found no expression of Alx3 in these animals (Fig. 5C). Additional evidence supporting reduced muscle innervation was indicated by decreased levels of ChAT and the presynaptic vesicle-associated protein synaptobrevin-2 (Fig. 5D and E).

We hypothesized that the observed effects in *Alx3*-deficient mice could be related to decreased sympathetic input regulating motor innervation at the neuromuscular junction [40, 41]. To test this, we determined the levels of markers of the presynaptic noradrenergic terminals in the gastrocnemius muscle. We found that the levels of noradrenaline-synthetizing enzyme tyrosine hydroxylase and the vesicular monoamine transporter 2 (VMAT2) were lower in *Alx3*-deficient than in control mice (Fig. 5D and E). In addition, in these muscles, we found decreased levels of noradrenaline (Fig. 5F). These results indicate the presence of decreased sympathetic innervation of muscles associated with *Alx3* deficiency.

The lack of expression of Alx3 in the spinal cord (see Fig. 5C) indicated that the observed alteration of the sympathetic innervation is not due to a direct effect of Alx3 deficiency in neurons of the intermediolateral cell column. Therefore, we tested for expression of Alx3 in the area containing the rostral ventrolateral medulla and the lateral paragigantocellular nucleus, two important brainstem centers controlling sympathetic output [42]. Noradrenergic neurons in these nuclei and in the nucleus tractus solitarius were readily identified using tyrosine hydroxylase immunostaining, but Alx3 immunolabeling was undetectable (Supplementary Fig. 8). Therefore, we argued that altered sympathetic outflow is likely due to a defect located within higher structures in the central nervous system.

### *Alx3*-deficient mice exhibit hypothalamic dysfunction and altered responses to fasting and feeding

**Fig. 6:**
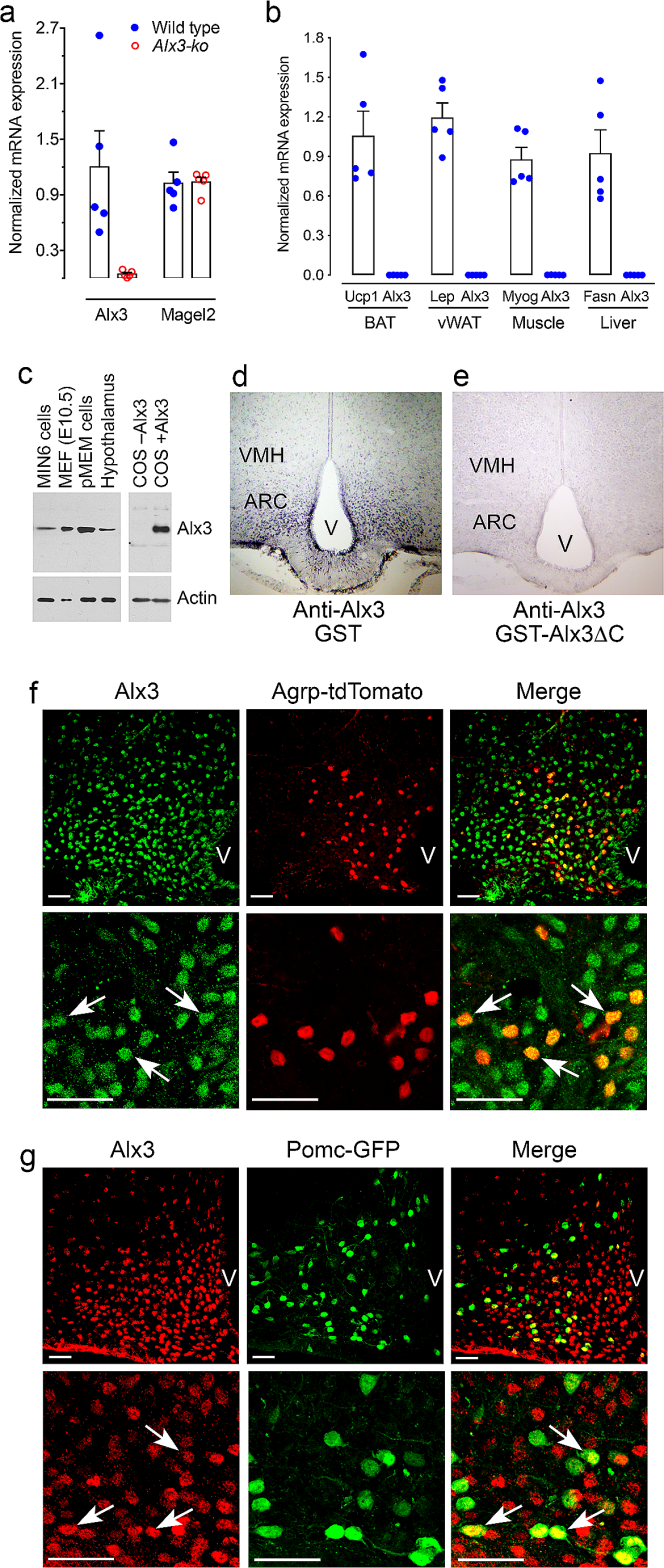
Expression of Alx3 in the hypothalamic arcuate nucleus (**A**) RT-qPCR showing expression of Alx3 mRNA in the hypothalamus of wild type but not Alx3-ko mice. Magel2 was used as a positive control (*n* = 5 per genotype). (**B**) RT-qPCR showing lack of expression of Alx3 mRNA in peripheral organs of wild type mice. Leptin (Lep), myogenin (Myo), fatty acid synthase (Fasn) and uncoupling protein 1 (Ucp1) were used as internal controls in each tissue. (**C**) Western blot showing expression of Alx3 in the mouse hypothalamus. As positive controls, lysates from MIN6 pancreatic β cells, mouse embryonic day 10.5 forebrain (MEF), primary mouse embryonic mesenchyme (pMEM) cells and COS cells transfected with an Alx3 expression plasmid were used. As a negative control, note the absence of Alx3 immunoreactivity in COS cells transfected with empty vector. (**D**) Representative coronal section of a wild type mouse brain showing Alx3-specific immunostaining enriched in the arcuate nucleus (ARC) and much weaker signal in the ventromedial hypothalamus (VMH). The antibody had been incubated in the presence of GST. (**E**) A similar section processed for immunohistochemistry using an Alx3 antibody pre-absorbed with GST-Alx3ΔC fusion protein. **F** and **G**) Confocal microscopy images, taken at low (upper panels) or high (lower panels) magnification, of coronal sections of brains from mice expressing Agrp-tdTomato (**F**) or Pomc-GFP (**G**) and labeled with an Alx3 antibody. Examples of arcuate Agrp or Pomc neurons co-expressing Alx3 are indicated by arrows. Scale bars represent 50 μm. V, third ventricle

Together, our data strongly suggest that the observed phenotype of *Alx3*-deficient mice is of central origin reflecting hypothalamic dysfunction. Therefore, we sought to determine whether *Alx3* is expressed in hypothalamic nuclei involved in the regulation of food intake and energy balance [43–45]. We detected the expression of *Alx3* mRNA in the basal hypothalamus of wild type but not in *Alx3*-deficient mice (Fig. 6A). The levels of *Magel2* mRNA, used as a positive control since it is prominently expressed in the arcuate nucleus and its deficiency results in phenotypic alterations with strong similarities to those observed in *Alx3*-deficient mice [46, 47], were not affected by *Alx3* deficiency (Fig. 6A). In wild type mice, expression of *Alx3* was not found in organs directly involved in peripheral metabolic regulation such as vWAT, BAT, muscle, or liver (Fig. 6B). In the hypothalamus, we confirmed the presence of Alx3 protein by western blot (Fig. 6C). Immunohistochemical staining revealed abundant Alx3-positive cells preferentially located within the arcuate nucleus (Fig. 6D). The specificity of the signal was confirmed by pre-absorption of the antibody (Fig. 6E and Supplementary Fig. 9) and by the absence of staining in other brain areas including the cortex, thalamus, and hippocampus (Supplementary Fig. 10). Immunofluorescence labeling confirmed co-expression of Alx3 in orexigenic NPY/AgRP neurons expressing *Agrp-tdTomato* (Fig. 6F) and in anorexigenic POMC/Cart neurons expressing *Pomc-GFP* (Fig. 6G).

We next investigated whether Alx3 expression in the hypothalamus is initiated during embryonic development. As a consequence of the targeting strategy used to delete the *Alx3* locus, which inserted a *lacZ* coding sequence in-frame in the *Alx3* exon 2 gene [6], *Alx3*-deficient mice express an Alx3-β-galactosidase fusion protein during embryonic development. Although it is transcriptionally inactive as it lacks part of the amino terminus, the DNA-binding homeodomain and the carboxyl terminus, this fusion protein retains an expression pattern practically identical to the Alx3 expression pattern observed in wild type animals [6, 7]. Taking advantage of this observation, we detected Alx3-β-galactosidase immunofluorescence in the hypothalamic primordium of E13.5 mouse embryos (Supplementary Fig. 11). A number of these cells also expressed ACTH, used as a marker for *Pomc* [48].

**Fig. 7:**
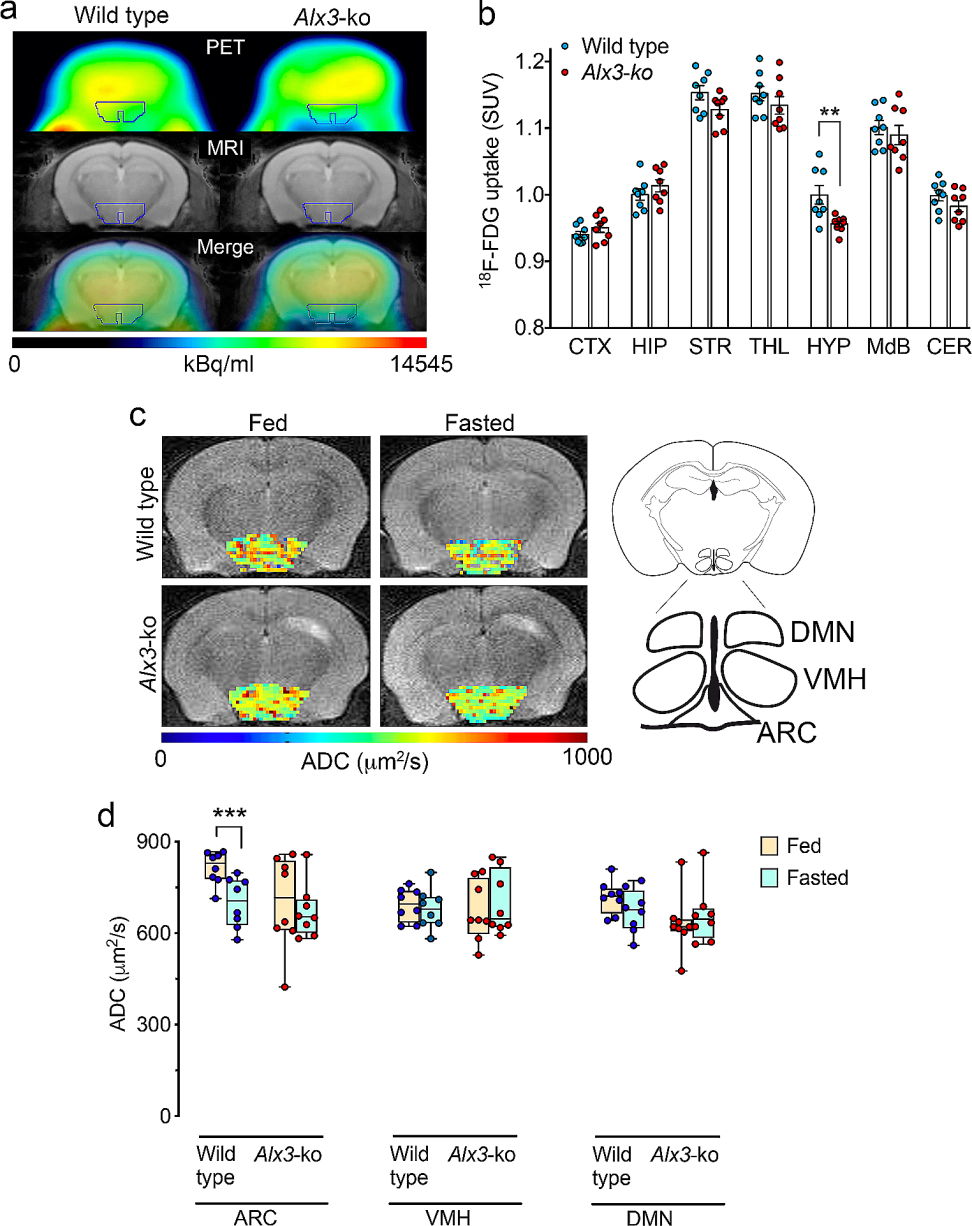
Alx3 deficient mice show altered hypothalamic function (**A**) Representative PET scan (upper panels), MRI template (middle panels) and merged (lower panels) images corresponding to coronal sections of mouse brains. The region of interest corresponding to the hypothalamic area used for quantification is indicated with a blue line. (**B**) Relative standard uptake value (SUV) of ^18^F-FDG detected in different brain regions relative to whole brain. CTX, cortex; HIP, hippocampus; STR, striatum; THL, thalamus; HYP, hypothalamus; MdB, midbrain; CER, cerebellum. **p < 0.01; Studentʼs *t*-test (*n* = 8 mice per genotype). (**C**) Representative axial images obtained by fDWI showing voxel apparent diffusion coefficient (ADC) intensity distribution in the hypothalamic area. The schematic depiction on the right panel represents the hypothalamic map indicating the relative location of the dorsomedial hypothalamic nucleus (DMN), ventromedial hypothalamus (VMH) and arcuate nucleus (ARC) regions from which fDWI measurements were taken. (**D**) Fitted apparent diffusion coefficient (ADC) values obtained from the arcuate nucleus (ARC), the ventromedial hypothalamus (VMH) or the dorsomedial nucleus (DMN) using fDWI in fed or fasted mice (blue dots, wild type; red dots, Alx3-ko). Box-and-whisker plots show median values, first and third quartiles, as well as maximum and minimum values. ***p < 0.001 (*n* = 8 per genotype)

Based on these results, we assessed whether Alx3 deficiency alters the metabolic activity of the ventral hypothalamus using PET. We found that the uptake of ^18^F-FDG was significantly reduced in the hypothalamus of *Alx3*-deficient mice, but not in any other cerebral region tested (Fig. 7A and B), suggesting that Alx3 is important for hypothalamic function. To confirm these results, we performed fDWI, which detects water molecule diffusion changes induced by neural activity in response to fasting with a higher degree of resolution in hypothalamic nuclei [24, 26] (Fig. 7C). We found that fasting resulted in lower diffusion coefficients in the arcuate nucleus of wild type mice, but the arcuate nucleus of *Alx3*-deficient animals was unresponsive (Fig. 7D). Furthermore, fasting had no effects on the ventromedial or dorsomedial hypothalamic nuclei of wild type or *Alx3*-deficient mice, indicating the specificity of the response (Fig. 7D). Because decreased diffusion coefficients are thought to be associated with cell swelling events concomitant to augmented neuronal activity [49, 50], the absence of detectable diffusion coefficient changes in the arcuate nucleus of *Alx3*-deficient mice led us to hypothesize the existence of hypothalamic dysfunction, suggesting that Alx3 is involved in the regulation of responses to fasting in this nucleus.

**Fig. 8:**
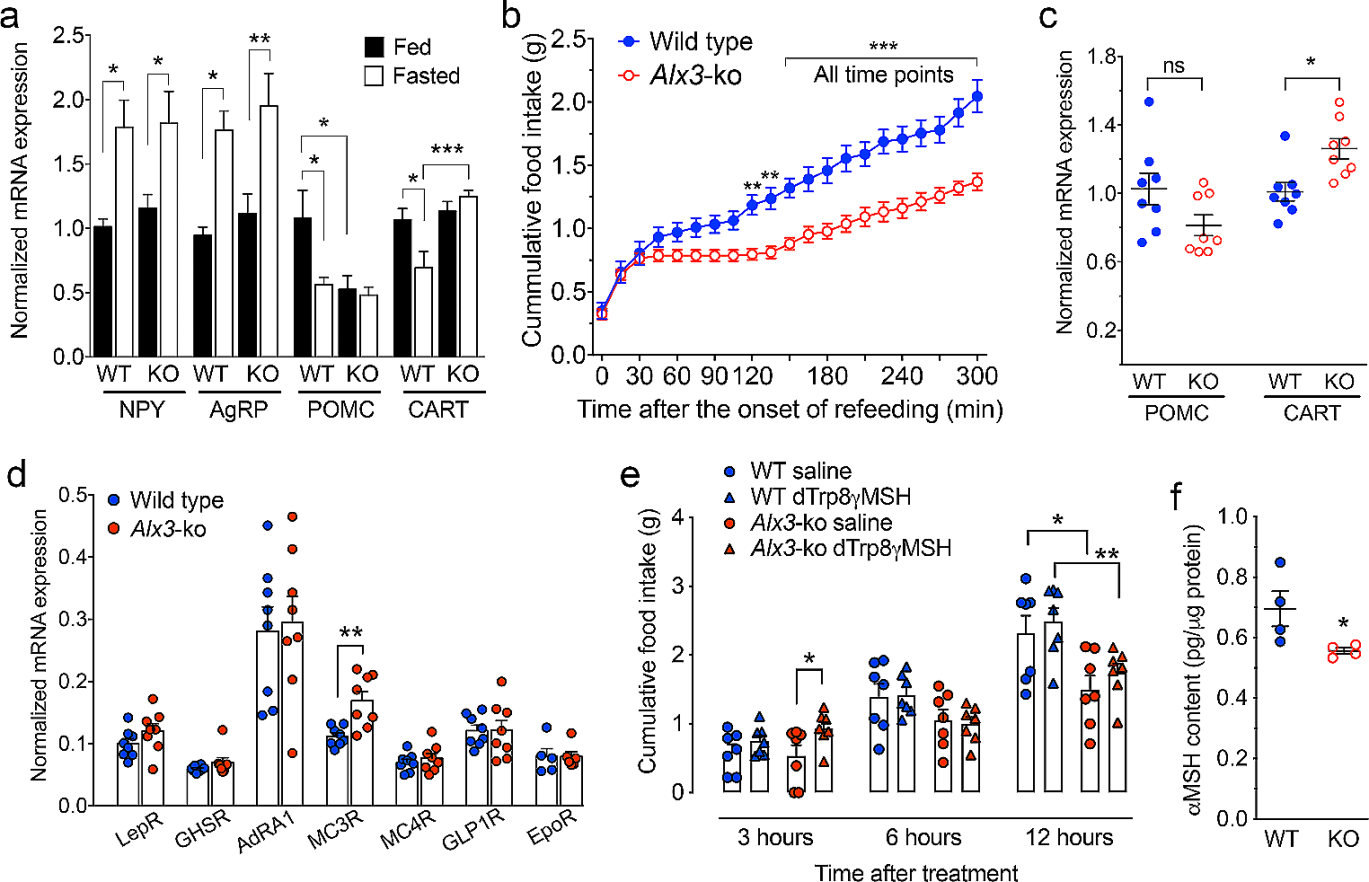
Altered food intake and gene expression in the arcuate nucleus of *Alx3-*deficient mice (**A**) Expression of orexigenic and anorexigenic genes in the arcuate nucleus of wild type (WT) or *Alx3*-deficient (KO) mice fed *ad libitum* or after an overnight fasting (*n* = 8 per genotype). (**B**) Cumulative quantification of food intake upon refeeding after an overnight fasting in mice (*n* = 14 wild type and 16 *Alx3*-deficient mice). (**C**) Expression of *Pomc* and *Cart* mRNA in the arcuate nucleus of wild type or *Alx3*-ko mice measured 5 h after the start of refeeding following an overnight fast (*n* = 8 per genotype). (**D**) Expression of receptor genes in the arcuate nucleus of wild type or *Alx3*-deficient mice fed *ad libitum*. Receptors for leptin (LepR), ghrelin (GHSR), adenosine (AdRA1), melanocortin (MC3R and MC4R), glucagon-like peptide 1 (GLP1R) and erythropoietin (EpoR) were tested (*n* = 8 per genotype except for EpoR, 5 wild type and 6 *Alx3*-deficient mice). (**E**) Cumulative measurement of the amount of food eaten after treatment with either saline or the MC3R agonist D-Trp8-γMSH (*n* = 7 per genotype). (F) Levels of αMSH in arcuate nucleus homogenates from wild type or *Alx3*-deficient mice (*n* = 4 per genotype). *p < 0.05, **p < 0.01 and ***p < 0.001; two-way ANOVA followed by Bonferroni transformation (**A**, **B** and **E**) or Student’s t test (C and D)

To address this question, we determined the expression of genes encoding orexigenic and anorexigenic peptides in the arcuate nucleus [51] in mice fed *ad libitum* or subjected to fasting overnight. We found that the increased expression of the orexigenic genes *Npy* and *Agrp* in response to fasting was similar in wild type and *Alx3*-deficient mice (Fig. 8A). In marked contrast, the expression of *Pomc* mRNA was lower in *Alx3-*deficient mice than in control animals fed *ad libitum*, and did not respond with the expected decrease upon fasting (Fig. 8A). The expression of *Cart* was similar in mice of both genotypes fed *ad libitum*, but contrary to what was observed in control animals, it did not respond with the expected decrease in *Alx3*-deficient mice after fasting (Fig. 8A). Thus, *Pomc/Cart*-dependent mechanisms regulating satiety are altered in *Alx3*-deficient mice.

To further investigate this, we determined the amount of food eaten upon re-feeding after an overnight fast. No differences in food intake were observed during the first 30 min of food availability (Fig. 8B), but then *Alx3*-deficient mice ceased eating for approximately two hours, whereas wild type mice went on feeding so that cumulative food intake increased progressively during this period (Fig. 8B). After this time, *Alx3*-deficient mice resumed feeding, although the amount of food intake was lower than that observed in control mice during the rest of the experiment (Fig. 8B). In line with this observation, *Cart* expression was higher in *Alx3*-deficient than in control mice at the end of this period, whereas *Pomc* expression was not significantly altered (Fig. 8C).

Since *Cart* mRNA expression is regulated by circulating leptin [52], we measured the levels of this hormone in the blood and found that they were not significantly different in *Alx3*-deficient and control mice (Supplementary Fig. 12A). In addition, STAT3 phosphorylation and expression of SOCS3 in the arcuate nucleus after leptin treatment were similar in both genotypes (Supplementary Fig. 12B-E), indicating no alterations in leptin function. Furthermore, we found no significant differences in the expression of the receptors for leptin, ghrelin (GHSR), adenosine A1 [53], α-MSH MC4R, glucagon-like peptide 1 (GLP1R) [54] or erythropoietin (EpoR) [55], but the expression of the α-MSH receptor MC3R [56, 57] was higher in *Alx3*-deficient than in control animals (Fig. 8D). We then tested the effect on food intake of D-Trp8-γMSH, a selective agonist of MC3R whose administration has been shown to stimulate feeding [16, 58, 59]. We found that at the dose used (0.2 mg/kg i.p.), this peptide did not affect wild type animals but transiently stimulated feeding in *Alx3*-deficient mice during the first three hours after treatment (Fig. 8E). This effect had disappeared 6 h after the onset of treatment, and after 12 h food intake in *Alx3*-deficient animals returned to values below those observed in control mice. Since the effect of D-Trp8-γMSH on food intake is transient and does not last for more than a few hours [58, 59], and the data in Fig. 8E represent cumulative food intake from the time of agonist administration, the differences observed at 12 h reflect the constitutive decrease in food intake of *Alx3*-deficient mice as compared with wild type animals observed in previous experiments (see Supplementary Fig. 1C and Fig. 2B) and in all likelihood are independent of the effect of the peptide at an earlier time. Thus, these results indicate increased sensitivity of *Alx3*-deficient mice to the transient effect of the MC3R agonist D-Trp8-γMSH, consistent with increased expression of MC3R. In a different set of experiments, we found decreased arcuate nucleus levels of αMSH associated with Alx3 deficiency (Fig. 8F). Thus, the observed increase in MC3R in the arcuate nucleus could reflect a compensatory mechanism to reduced levels of αMSH.

## Discussion

Our work demonstrates the selective expression of Alx3 in the arcuate nucleus, which plays a fundamental role in the hypothalamic regulation of feeding and energy expenditure by integrating nutritional and hormonal signals from the periphery. Thus, the arcuate nucleus contributes to the maintenance of energy balance depending on the metabolic needs of the organism [13, 45]. Fittingly, we show that Alx3 deficiency correlates with altered energy homeostasis, reduced food intake, and decreased muscle mass at the expense of increased adiposity.

Our previous studies on the function of Alx3 in pancreatic islet cells indicated a role in the regulation of insulin and glucagon gene expression in a glucose-dependent manner [1, 4]. Using *Alx3*-deficient mice on a hybrid FVBxC57BL/6J background we found altered glucose homeostasis due to dysfunctional pancreatic islets resulting in mild hyperglycemia, glucose intolerance and impaired insulin secretion [5]. Since blood glucose fluctuations and insulin can affect food intake and lipid deposition, it is possible that pancreatic dysfunction may contribute to the phenotype described in this study. However, if present, that contribution would be expected to be small, because on a pure C57BL/6J background *Alx3*-deficient mice retain the reduced food intake and decreased muscle mass phenotype in the absence of altered body weight, but exhibit normal glycemic regulation indicating unaffected pancreatic function. The finding that *Alx3*-deficiency does not affect pancreatic islet function in C57BL/6J mice is not entirely unexpected, since it is well established that metabolic alterations in response to specific genetic manipulations or to submission to hypercaloric diet regimes can differ widely depending on the genetic background of the strain used in each case [60, 61].

On the other hand, the undetected expression of *Alx3* in major peripheral organs directly involved in metabolic regulation indicates that a primary dysfunction in these organs is unlikely to generate the observed phenotype. Rather, the presence of Alx3 in neurons of the arcuate nucleus and the existence of systemic metabolic dysfunctions associated with altered hypothalamic responses to fasting and refeeding in *Alx3*-deficient mice likely reflects an important role of this transcription factor in the hypothalamus.

As hypothalamic *Alx3*-deficiency appears to affect primarily the melanocortin system, the reduced food intake observed in mutant mice could be related to a premature response to induce satiety, as suggested by dysregulated *Pomc/Cart* expression upon fasting and abnormally increased *Cart* expression upon refeeding. Normally, *Pomc/Cart* neurons respond to leptin generated from the adipose tissue upon feeding [16], and as a consequence, they release αMSH to activate MC4R expressed in the paraventricular nucleus to promote satiety signals [62], to which co-released CART also contributes. However, the premature satiety response observed in *Alx3*-deficient mice appears to be unrelated to differences in the circulating levels of leptin or to altered activation of leptin receptors in the hypothalamus as assessed by STAT3 phosphorylation and SOCS3 expression, since they were similar in wild type and *Alx3*-deficient mice.

The importance of the arcuate nucleus POMC system in the control of appetite and satiety is indicated by experimental and clinical observations showing that deficits in some of its components are associated with obesity [63–65]. In this regard, some of the main features of the *Alx3*-deficient mice phenotype including increased adiposity, decreased lean mass and decreased food intake are similar to those observed in mice deficient for *Magel2*, a gene whose mutations are associated with Prader-Willi syndrome in humans [46]. However, although the *Magel2*-deficient phenotype is also associated with hypothalamic Pomc neuron dysfunction [47], it is unlikely that both phenotypes are directly related since we did not detect altered hypothalamic expression of *Magel2* in *Alx3*-deficient mice.

An important observation in our study is the increased expression of hypothalamic MC3R but not MC4R. Both receptors, stimulated by αMSH synthesized in *Pomc* neurons, are key components of the central circuits regulating food intake and energy expenditure [66]. MC4R, predominately expressed in the paraventricular nucleus that receives projections from the ARC, is known to regulate food intake, body weight, and energy homeostasis, and consistently, lack of function of this receptor in mice and humans results in hyperphagia and obesity [66]. In turn, MC3R is primarily expressed in the ventromedial hypothalamus and in the arcuate nucleus, where it is present in pre- and post-synaptic terminals modulating the activity of *Pomc/Cart* neurons via autoinhibitory feed-back loops [16, 67]. The enriched expression of Alx3 in the arcuate nucleus of control animals, and the altered cellular activity in response to fasting detected by fDWI only in this nucleus in *Alx3*-deficient mice, suggest that the decreased levels of αMSH and the elevated expression of MC3R in *Alx3*-deficient mice reflects a role of Alx3 in the regulation of the intrinsic *Pomc/Cart*-*NPY/AgRP* circuitry.

Remarkably, the phenotype observed in *Alx3*-deficient mice shares key features with the phenotype of *Mc3r*-deficient mice, including increased adiposity without a proportional increase in body weight, reduced lean mass, and normal or decreased food intake [56, 57]. An additional similarity is that both *Alx3*- and *Mc3r-*deficient mice show reduced energy expenditure and RER [56], indicating the utilization of a higher proportion of lipids as a source of fuel. The comparison between both phenotypes is relevant because both are consistent with decreased αMSH-dependent signaling in the hypothalamus, the former due to reduced levels of the ligand, and the latter due to absence of receptors.

Despite these striking similarities, we observed that *Alx3*-deficient mice exhibit increased expression of *Mc3r* in the hypothalamus, and confirmed that these receptors are functional as indicated by the transient increase in food intake observed after administration of the selective MC3R agonist D-Trp8-γMSH, consistent with previous observations [58, 59]. Nonetheless, as MC3R is stimulated by αMSH and antagonized by AgRP, a possible reduction in the levels of αMSH as a consequence of decreased *Pomc* expression could contribute to decreased function of this receptor. Indeed, the elevated levels of MC3R mRNA expression and increased sensitivity to D-Trp8-γMSH in *Alx3*-deficient animals could reflect a compensatory mechanism to reduced αMSH content detected in the arcuate nucleus.

Studying the effects of the D-Trp8-γMSH agonist on arcuate nucleus neurons, Cowley et al. [16] proposed that MC3R acts as a negative feed-back autoreceptor to decrease POMC neuronal activity in response to elevated αMSH, a notion also supported by more recent studies [67]. Accordingly, the transient increase in food intake observed in our own and other studies after administration of D-Trp8-γMSH could be interpreted as the consequence of transiently decreased satiety signals due to autoreceptor-induced inhibition of *Pomc* neuron activity. Nonetheless, given the complexity of the hypothalamic neuronal circuits involving activation and blockade of αMSH receptors located both at pre- and post-synaptic levels, the demonstration of a primary involvement of MC3R in the phenotype of *Alx3*-deficient animals awaits further investigations, and the involvement of other currently unidentified regulatory mechanisms cannot be excluded at the present time. Of note, the predominant function of MC3R appears to be associated with the regulation of energy rheostasis, rather than with the control of food intake, by contributing to the adjustment of homeostatic set points affecting energy intake and storage to adapt the organism to changing metabolic requirements [67, 68].

The changes in adiposity and muscle mass independently of decreased food intake suggest a role for Alx3 in the central regulation of peripheral nutrient partitioning, possibly involving modulation of the activity of the sympathetic nervous system as suggested in the case of melanocortin receptors [65, 69]. Sympathetic activity regulated by the hypothalamic POMC system promotes lipolysis and glucose uptake in white and brown adipose tissues, respectively [69, 70]; enhances hepatic glucose production and glucose uptake in muscle [71, 72], and modulates pancreatic islet function by affecting insulin and glucagon secretion [72]. The higher levels of expression of lipolytic genes and the relatively reduced increment in the accumulation of visceral fat in HFD-fed *Alx3*-deficient mice are in agreement with increased sympathetic tone [30], a notion further supported by the observed increase of tyrosine hydroxylase levels and noradrenaline content in the vWAT of these mice.

Although no differences in the expression of lipolytic or lipogenic genes were found between *Alx3*-deficient and control mice fed with SCD, a significant increase in the expression of *Pck1* in the adipose tissue of mutant animals was observed. PCK1 is the rate-limiting enzyme of glyceroneogenesis for the synthesis of triacylglycerol in the adipose tissue. Consequently, overexpression of *Pck1* in adipocytes results in obesity [29, 73]. In vWAT, the activity of PCK1 is directly regulated by the sympathetic tone, as assessed by reduced levels of triacylglycerol in adipocytes after sympathectomy [74]. Thus, increased fat mass in SCD-fed *Alx3*-deficient mice is consistent with increased levels of noradrenaline and enhanced expression of tyrosine hydroxylase, indicating increased sympathetic tone, as well as with enhanced expression of *Pck1.* Despite having a higher fat proportion under SCD, *Alx3*-deficient mice accumulate proportionally less fat when fed under a HFD regime. A possible explanation for this observation is that, when submitted to a HFD regime, the induced expression of the lipogenic gene *Fasn* in *Alx3*-deficent mice is lower relative to the induction observed in wild type mice, whereas that of the lipolytic genes *Hsl* and *Atgl* is higher. Thus, under a HFD, in *Alx3*-ko mice lipogenesis would be lower than in wild type animals, whereas lipolysis would be higher, and as a consequence fat accumulation would be expected to be, in relative terms, lower in *Alx3*-ko than in wild type mice.

Interestingly, glucose tolerance and insulin sensitivity improved in *Alx3*-deficient mice subjected to HFD despite similar levels of inflammatory markers in the adipose tissue as compared with wild type animals, although the existence of a direct association between inflammation and insulin resistance has recently been put into question [75]. This apparently paradoxical response may be related to the higher vWAT adiponectin expression in *Alx3*-deficient mice fed under a HFD regime as compared with similarly fed wild type mice [76], and is in agreement with the observed increase in the expression of genes associated with hepatic glucose uptake and glycogenesis.

Wild type and *Alx3*-decicient mice accumulated similar absolute amounts of adipose tissue when fed with HFD. Despite having the same amount of fat, *Alx3*-deficient mice showed lower body weight than wild type animals when both were fed with a HFD. Thus, the content of accumulated fat is unlikely to account for these differences in body weight. Also, the absence of differences in the measurements of the tibia indicates that body length is unlikely to account for differences in body weight either. Thus, a possible explanation for the lower body weight in Alx3-deficient mice relative to control animals fed with HFD is likely to be related to the decreased muscle mass observed in these animals.

The lower energy expenditure observed in *Alx3*-deficient mice is also consistent with the reduced muscle mass [77, 78]. Lower energy expenditure in mutant mice was observed during the entire 24 h daily cycle when fed with HFD, whereas when fed under a SCD regime, lower energy expenditure was observed predominantly during the night phase coinciding with increased locomotor activity or during forced exercise on a treadmill. These observations are in agreement with studies showing that energy expenditure is directly correlated with the amount of muscle mass, and that decreased muscle mass is associated with lower energy expenditure and increased visceral fat accumulation [77–79]. Thus, the relatively higher accumulation of fat observed in *Alx3*-deficient mice under SCD conditions could be a consequence of primarily reduced muscle mass.

Since the expression of *Alx3* was undetectable in muscle, a primary myogenic defect is unlikely to be responsible for decreased muscle mass in *Alx3*-deficient mice, consistent with the absence of significant differences in the expression of endogenous muscle trophic factors between both genotypes. Instead, our data strongly suggests that reduced muscle mass is associated with denervation. Whether motor neuron dysfunction plays a primary role in neuromuscular junction denervation in *Alx3*-deficient mice cannot be completely ruled out at this time, although the undetectable levels of *Alx3* expression in the spinal cord indicates that denervation was not due to a direct effect of Alx3 deficiency in motor neurons. Nonetheless, electrophysiological recordings could be used to test motor neuron activity in the anterior horn of the spinal cord.

We favor the hypothesis that the reduced muscle mass in *Alx3*-deficient mice may be related to the possible existence of altered sympathetic output, since evidence for an important role of the sympathetic nervous system in the maintenance of skeletal muscle mass and function has been accumulating in recent years [80, 81]. The sympathetic innervation reaches the neuromuscular junction and is crucial for the maintenance of motor function, to the extent that selective skeletal muscle sympathectomy results in muscle weakness, impaired cholinergic neurotransmission and fragmentation and dispersion of the AChR clusters [40, 41, 82, 83]. The recent introduction of sympathomimetic drugs to treat chronic myasthenic syndromes characterized by muscle weakness and fatigue in humans further supports the importance of sympathetic stimulation of the neuromuscular junction for adequate function [84, 85]. Furthermore, in vivo experiments have demonstrated that postganglionic sympathetic neurons directly regulate neuromuscular transmission by activating β1-adrenergic receptors expressed in motoneuron axonal terminals [86].

The gastrocnemius is one of a number of skeletal muscles for which a direct innervation of the neuromuscular junction by sympathetic fibers has been demonstrated [41, 82]. The existence of reduced sympathetic innervation in the gastrocnemius muscle of *Alx3*-deficient mice is indicated by reduced noradrenaline content as well as by decreased levels of tyrosine hydroxylase and VMAT2, two proteins present in sympathetic nerve endings. In addition, the existence of reduced muscle innervation is supported by decreased levels of Syb-2, a presynaptic vesicle marker. As Syb-2 is a general component of the SNARE complex mediating vesicle exocytosis from nerve terminals, reduced gastrocnemius innervation may involve both sympathetic and motor neuron terminals, as attested by the observed decrease in the levels of ChAT, a protein that is specific for cholinergic neurons. Finally, reduced muscle strength and exercise performance as well as increased expression of myogenin, AChR subunits and AChE, considered as markers of decreased cholinergic neurotransmission [32, 34, 87], are also consistent with decreased skeletal muscle innervation in *Alx3*-deficient mice.

In humans, since the original identification of *ALX3* by Twigg et al. [9] as the causative gene for a characteristic form of congenital frontonasal dysplasia, it has been described that this disease can include intellectual disability, ankyloglossia, hearing loss, and agenesis of the corpus callosum. To date, no metabolic alterations have been reported in these patients. However, it is important to bear in mind that *ALX3*-dependent frontonasal dysplasia is a rare disease with only a relatively small number of cases reported, and that patients are usually children within the first few years of life.

## Limitations of the study

Our data indicate that Alx3 coordinately regulates food intake, energy expenditure and nutrient partitioning affecting adiposity and muscle mass. We identify the arcuate nucleus as a potential site from which Alx3 exerts at least some of these functions. Although Alx3 expression in adult tissues appears to be relatively restricted to discrete locations, the use of a constitutive knock out model represents a limitation, and the confirmation of a specific role for Alx3 in the regulation of Agrp and Pomc neurons in the arcuate nucleus awaits neuron-specific inactivation of the *Alx3* gene. As our study was carried out using only male mice, it will be important to determine whether a similar phenotype is observed in females.

An important question that remains to be addressed is the exact mechanism by which MC3R expression is increased in Alx3-deficient mice, as alterations in the function of this important receptor, key for the function of the internal arcuate nucleus neuronal circuitry, have important consequences for the regulation of food intake and energy homeostasis. Since the MC3R receptors act predominantly regulating feed-back loops between Agrp and Pomc neurons with the arcuate nucleus, this question will require specific approaches to determine their functions relative to their specific neuronal type and location.

Finally, another important question that remains to be addressed in future studies is the origin of the decreased muscle mass and the adipose tissue disbalance. Although our data support the involvement of dysfunctional sympathetic innervation of these tissues, the contribution of a developmental defect possibly affecting myogenesis during embryonic development cannot be entirely ruled out, since the importance of the transient expression of Alx3 in previously unrecognized discrete embryonic cell types is starting to emerge [88].

Despite these limitations, since the contribution of dysfunctional nutrient partitioning to the development of obesity and diabetes remains poorly understood, our work opens new avenues for the study of the pathophysiology of these metabolic disorders.

### Electronic supplementary material

Below is the link to the electronic supplementary material.

Supplementary Material 1

## Data Availability

The data that support the findings of this study are available from the corresponding author upon request. Data will be deposited in the institutional CSIC repository.

## References

[1] Mirasierra M, Vallejo M (2006) The homeoprotein Alx3 expressed in pancreatic β-cells regulates insulin gene transcription by interacting with the basic helix-loop-helix protein E47. Mol Endocrinol 20:2876–2889. 10.1210/me.2005-0472 16825292 10.1210/me.2005-0472

[2] Fernández-Pérez A, Vallejo M (2014) Pdx1 and USF transcription factors co-ordinately regulate Alx3 gene expression in pancreatic β-cells. Biochem J 463:287–296. 10.1042/BJ20140643 25040025 10.1042/BJ20140643

[3] García-Sanz P, Mirasierra M, Vallejo M (2017) Embryonic defence mechanisms against glucose-dependent oxidative stress require enhanced expression of Alx3 to prevent malformations during diabetic pregnancy. Sci Rep 7:389. 10.1038/s41598-017-00334-1 28341857 10.1038/s41598-017-00334-1 PMC5428206

[4] Mirasierra M, Vallejo M (2016) Glucose-dependent downregulation of glucagon gene expression mediated by selective interactions between ALX3 and PAX6 in mouse alpha cells. Diabetologia 59:766–775. 10.1007/s00125-015-3849-4 26739814 10.1007/s00125-015-3849-4

[5] Mirasierra M, Fernández-Pérez A, Díaz-Prieto N, Vallejo M (2011) Alx3-deficient mice exhibit decreased insulin in beta cells, altered glucose homeostasis and increased apoptosis in pancreatic islets. Diabetologia 54:403–414. 10.1007/s00125-010-1975-6 21104068 10.1007/s00125-010-1975-6

[6] Beverdam A, Brouwer A, Reijnen M, Korving J, Meijlink F (2001) Severe nasal clefting and abnormal embryonic apoptosis in Alx3/Alx4 double mutant mice. Development 128:3975–3986. 10.1242/dev.128.20.3975 11641221 10.1242/dev.128.20.3975

[7] Lakhwani S, Garcia-Sanz P, Vallejo M (2010) Alx3-deficient mice exhibit folic acid-resistant craniofacial midline and neural tube closure defects. Dev Biol 344:869–880. 10.1016/j.ydbio.2010.06.002 20534379 10.1016/j.ydbio.2010.06.002

[8] Mallarino R, Henegar C, Mirasierra M et al (2016) Developmental mechanisms of stripe patterns in rodents. Nature 359:518–523. 10.1038/nature20109 10.1038/nature20109 PMC5292240 27806375

[9] Twigg SRF, Versnel SL, Nurnberg G et al (2009) Frontorhiny, a distinctive presentation of frontonasal dysplasia caused by recessive mutations in the ALX3 homeobox gene. Am J Hum Genet 84:1–8. 10.1016/j.ajhg.2009.04.009 10.1016/j.ajhg.2009.04.009 PMC2681074 19409524

[10] García MC, Wernstedt I, Berndtsson A et al (2006) Mature-onset obesity in interleukin-1 receptor I knockout mice. Diabetes 55:1205–1213. 10.2337/db05-1304 16644674 10.2337/db05-1304

[11] Tang H, Vasselli JR, Wu EX, Boozer C, Gallagher D (2002) High-resolution magnetic resonance imaging tracks changes in organ and tissue mass in obese and aging rats. Am J Physiol Regul Integr Comp Physiol 282:R890–R899. 10.1152/ajpregu.0527.2001 11832412 10.1152/ajpregu.0527.2001

[12] Solís O, García-Montes JR, García-Sanz P et al (2017) Human COMT over-expression confers a heightened susceptibility to dyskinesia in mice. Neurobiol Dis 102:133–139. 10.1016/j.nbd.2017.03.006 28315782 10.1016/j.nbd.2017.03.006 PMC5481205

[13] Del Río-Martín A, Pérez-Taboada I, Fernandez-Pérez A, Moratalla R, De la Villa P, Vallejo M (2019) Hypomorphic expression of Pitx3 disrupts circadian clocks and prevents metabolic entrainment of Energy Expenditure. Cell Rep 29:3678–3692. 10.1016/j.celrep.2019.11.027 31825844 10.1016/j.celrep.2019.11.027

[14] Perez LJ, Rios L, Trivedi P et al (2017) Validation of optimal reference genes for quantitative real time PCR in muscle and adipose tissue for obesity and diabetes research. Sci Rep 7:3612. 10.1038/s41598-017-03730-9 28620170 10.1038/s41598-017-03730-9 PMC5472619

[15] Livak K, Schmittgen TD (2001) Analysis of relative gene expression data using real-time quantitative PCR and the 2^–∆∆C^T method. Methods 25:402–408. 10.1006/meth.2001.1262 11846609 10.1006/meth.2001.1262

[16] Cowley MA, Smart JL, Rubinstein M et al (2001) Leptin activates anorexigenic POMC neurons through a neural network in the arcuate nucleus. Nature 411:480–484. 10.1038/35078085 11373681 10.1038/35078085

[17] Estañ MC, Fernández-Núñez E, Zaki MS et al (2019) Recessive mutations in muscle-specific isoforms of FXR1 cause congenital multi-minicore myopathy. Nat Commun 10:797. 10.1038/s41467-019-08548-9 30770808 10.1038/s41467-019-08548-9 PMC6377633

[18] Benoit B, Meugnier E, Castelli M et al (2017) Fibroblast growth factor 19 regulates skeletal muscle mass and ameliorates muscle wasting in mice. Nat Med 23:990–996. 10.1038/nm.4363 28650457 10.1038/nm.4363

[19] Molinero A, Fernandez-Pérez A, Mogas A et al (2017) Role of muscle IL-6 in gender-specific metabolism in mice. PLoS ONE 12:e0173675. 10.1371/journal.pone.0173675 28319140 10.1371/journal.pone.0173675 PMC5358764

[20] García-Sanz P, Fernández-Pérez A, Vallejo M (2013) Differential configurations involving binding of USF transcription factors and Twist1 regulate Alx3 promoter activity in mesenchymal and pancreatic cells. Biochem J 450:199–208. 10.1042/BJ20120962 23181698 10.1042/BJ20120962

[21] Cantó C, Suárez E, Lizcano JM et al (2004) Neuregulin signaling on glucose transport in muscle cells. J Biol Chem 279:12260–12268. 10.1074/jbc.M308554200 14711829 10.1074/jbc.M308554200

[22] Steculorum SM, Paeger L, Bremser S et al (2015) Hypothalamic UDP increases in obesity and promotes feeding via P2Y6-Dependent activation of AgRP neurons. Cell 162:1404–1417. 10.1016/j.cell.2015.08.032 26359991 10.1016/j.cell.2015.08.032

[23] Ma Y, Smith D, Hof PR et al (2008) In vivo 3D digital atlas database of the adult C57BL/6J mouse brain by magnetic resonance microscopy. Front Neuroanat 2:1. 10.3389/neuro.05.001.2008 18958199 10.3389/neuro.05.001.2008 PMC2525925

[24] Lizarbe B, Benítez A, Sánchez-Montañés M et al (2013) Imaging hypothalamic activity using diffusion weighted magnetic resonance imaging in the mouse and human brain. NeuroImage 64:448–457. 10.1016/j.neuroimage.2012.09.033 23000787 10.1016/j.neuroimage.2012.09.033

[25] Franklin KBJ, Paxinos G (2008) The mouse brain in stereotaxic coordinates. Elsevier, Amsterdam

[26] Lizarbe B, Fernández-Pérez A, Caz V et al (2019) Systemic Glucose Administration Alters Water Diffusion and Microvascular Blood Flow in mouse hypothalamic nuclei - an fMRI study. Front Neurosci 13:921. 10.3389/fnins.2019.00921 31551685 10.3389/fnins.2019.00921 PMC6733885

[27] Rubio WB, Cortopassi MD, Banks AS (2023) Indirect calorimetry to assess Energy Balance in mice: Measurement and Data Analysis. Methods Mol Biol 2662:103–115. 10.1007/978-1-0716-3167-6_9 37076674 10.1007/978-1-0716-3167-6_9

[28] Campillo BW, Galguera D, Cerdán S, López-Larrubia P, Lizarbe B (2022) Short-term high-fat diet alters the mouse brain magnetic resonance imaging parameters consistently with neuroinflammation on males and metabolic rearrangements on females. A pre-clinical study with an optimized selection of linear mixed-effects models. Front NeuroSci 16:1025108. 10.3389/fnins.2022.1025108 36507349 10.3389/fnins.2022.1025108 PMC9729798

[29] Yu S, Meng S, Xiang M, Ma H (2021) Phosphoenolpyruvate carboxykinase in cell metabolism: roles and mechanisms beyond gluconeogenesis. Mol Metabolism 53:101257. 10.1016/j.molmet.2021.101257 10.1016/j.molmet.2021.101257 PMC8190478 34020084

[30] Zeng W, Pirzgalska RM, Pereira MM et al (2015) Sympathetic neuro-adipose connections mediate leptin-driven lipolysis. Cell 163:84–94. 10.1016/j.cell.2015.08.055 26406372 10.1016/j.cell.2015.08.055 PMC7617198

[31] Hatori M, Vollmers C, Zarrinpar A et al (2012) Time-restricted feeding without reducing caloric intake prevents metabolic diseases in mice fed a high-fat diet. Cell Metab 15:848–860. 10.1016/j.cmet.2012.04.019 22608008 10.1016/j.cmet.2012.04.019 PMC3491655

[32] Vaughan SK, Sutherland NM, Valdez G (2019) Attenuating Cholinergic Transmission increases the number of Satellite cells and preserves muscle Mass in Old Age. Front Aging Neurosci 11:262. 10.3389/fnagi.2019.00262 31616286 10.3389/fnagi.2019.00262 PMC6768977

[33] Moresi V, Williams AH, Meadows E et al (2010) Myogenin and class II HDACs control neurogenic muscle atrophy by inducing E3 ubiquitin ligases. Cell 143:35–45. 10.1016/j.cell.2010.09.004 20887891 10.1016/j.cell.2010.09.004 PMC2982779

[34] Méjat A, Ramond F, Bassel-Duby R, Khochbin S, Olson EN, Schaeffer L (2005) Histone deacetylase 9 couples neuronal activity to muscle chromatin acetylation and gene expression. Nat Neurosci 8:313–321. 10.1038/nn1408 15711539 10.1038/nn1408

[35] Henderson CE, Phillips HS, Pollock RA et al (1994) GDNF: a potent survival factor for motoneurons present in peripheral nerve and muscle. Science 266:1062–1064. 10.1126/science.7973664 7973664 10.1126/science.7973664

[36] Stanga S, Brambilla L, Tasiaux B et al (2018) A role for GDNF and Soluble APP as biomarkers of amyotrophic lateral sclerosis pathophysiology. Front Neurol 9:384. 10.3389/fneur.2018.00384 29899726 10.3389/fneur.2018.00384 PMC5988896

[37] Baczek J, Silkiewicz M, Wojszel ZB (2020) Myostatin as a biomarker of muscle wasting and other pathologies-State of the art and knowledge gaps. Nutrients 12:E2401. 10.3390/nu12082401 10.3390/nu12082401 PMC7469036 32796600

[38] Kim JK, Jha NN, Feng Z et al (2020) Muscle-specific SMN reduction reveals motor neuron-independent disease in spinal muscular atrophy models. J Clin Invest 130:1271–1287. 10.1172/JCI131989 32039917 10.1172/JCI131989 PMC7269591

[39] Alkaslasi MR, Piccus ZE, Hareendran S et al (2021) Single nucleus RNA-sequencing defines unexpected diversity of cholinergic neuron types in the adult mouse spinal cord. Nat Commun 12:2471. 10.1038/s41467-021-22691-2 33931636 10.1038/s41467-021-22691-2 PMC8087807

[40] Khan MM, Lustrino D, Silveira WA et al (2016) Sympathetic innervation controls homeostasis of neuromuscular junctions in health and disease. Proc Natl Acad Sci U S A 113:746–750. 10.1073/pnas.1524272113 26733679 10.1073/pnas.1524272113 PMC4725522

[41] Rodrigues ACZ, Messi ML, Wang ZM et al (2019) The sympathetic nervous system regulates skeletal muscle motor innervation and acetylcholine receptor stability. Acta Physiol 225:e13195. 10.1111/apha.13195 10.1111/apha.13195 PMC7224611 30269419

[42] Lorenzo-Martín LF, Menacho-Márquez M, Fabbiano S et al (2019) Vagal afferents contribute to sympathoexcitation-driven metabolic dysfunctions. J Endocrinol 240:483–496. 10.1530/JOE-18-0623 30703063 10.1530/JOE-18-0623 PMC6368248

[43] Krashes MJ, Shah BP, Madara JC et al (2014) An excitatory paraventricular nucleus to AgRP neuron circuit that drives hunger. Nature 507:238–242. 10.1038/nature12956 24487620 10.1038/nature12956 PMC3955843

[44] Orozco-Solís R, Aguilar-Arnal L, Murakami M et al (2016) The circadian clock in the ventromedial hypothalamus controls cyclic energy expenditure. Cell Metab 23:467–478. 10.1016/j.cmet.2016.02.003 26959185 10.1016/j.cmet.2016.02.003 PMC5373494

[45] Schneeberger M, Gomis R, Claret M (2014) Hypothalamic and brainstem neuronal circuits controlling homeostatic energy balance. J Endocrinol 220:T25–T46. 10.1530/JOE-13-0398 24222039 10.1530/JOE-13-0398

[46] Bischof JM, Stewart CL, Wevrick R (2007) Inactivation of the mouse Magel2 gene results in growth abnormalities similar to Prader-Willi syndrome. Hum Mol Genet 16:2713–2719. 10.1093/hmg/ddm225 17728320 10.1093/hmg/ddm225

[47] Oncul M, Dilsiz P, Oz EA et al (2018) Impaired melanocortin pathway function in Prader-Willi syndrome gene-Magel2 deficient mice. Hum Mol Genet 27:3129–3136. 10.1093/hmg/ddy216 29878108 10.1093/hmg/ddy216

[48] Orquera DP, Tavella MB, de Souza FSJ, Nasif S, Low MJ, Rubinstein M (2019) The Homeodomain transcription factor NKX2.1 is essential for the early specification of Melanocortin Neuron Identity and activates Pomc expression in the developing hypothalamus. J Neurosci 39:4023–4035. 10.1523/jneurosci.2924-18.2019 30886014 10.1523/jneurosci.2924-18.2019 PMC6529873

[49] Jin T, Kim SG (2008) Functional changes of apparent diffusion coefficient during visual stimulation investigated by diffusion-weighted gradient-echo fMRI. NeuroImage 41:801–812. 10.1016/j.neuroimage.2008.03.014 18450483 10.1016/j.neuroimage.2008.03.014 PMC2527868

[50] Abe Y, Tsurugizawa T, Le Bihan D (2017) Water diffusion closely reveals neural activity status in rat brain loci affected by anesthesia. PLoS Biol 15:e2001494. 10.1371/journal.pbio.2001494 28406906 10.1371/journal.pbio.2001494 PMC5390968

[51] Williams KW, Elmquist JK (2012) From neuroanatomy to behavior: central integration of peripheral signals regulating feeding behavior. Nat Neurosci 15:1350–1355. 10.1038/nn.3217 23007190 10.1038/nn.3217 PMC3763714

[52] Lau J, Herzog H (2014) CART in the regulation of appetite and energy homeostasis. Front Neurosci 8:313. 10.3389/fnins.2014.00313 25352770 10.3389/fnins.2014.00313 PMC4195273

[53] Yang L, Qi Y, Yang Y (2015) Astrocytes control food intake by inhibiting AGRP neuron activity via adenosine A1 receptors. Cell Rep 11:798–807. 10.1016/j.celrep.2015.04.002 25921535 10.1016/j.celrep.2015.04.002

[54] Secher A, Jelsing J, Baquero AF et al (2014) The arcuate nucleus mediates GLP-1 receptor agonist liraglutide-dependent weight loss. J Clin Invest 124:4473–4488. 10.1172/JCI75276 25202980 10.1172/JCI75276 PMC4215190

[55] Teng R, Gavrilova O, Suzuki N et al (2011) Disrupted erythropoietin signalling promotes obesity and alters hypothalamus proopiomelanocortin production. Nat Commun 2:250. 10.1038/ncomms1526 22044999 10.1038/ncomms1526 PMC3542973

[56] Butler AA, Kesterson RA, Khong K et al (2000) A unique metabolic syndrome causes obesity in the melanocortin-3 receptor-deficient mouse. Endocrinology 141:3518–3521. 10.1210/endo.141.9.7791 10965927 10.1210/endo.141.9.7791

[57] Chen AS, Marsh DJ, Trumbauer ME et al (2000) Inactivation of the mouse melanocortin-3 receptor results in increased fat mass and reduced lean body mass. Nat Genet 26:97–102. 10.1038/79254 10973258 10.1038/79254

[58] Marks DL, Hruby V, Brookhart G, Cone RD (2006) The regulation of food intake by selective stimulation of the type 3 melanocortin receptor (MC3R). Peptides 27:259–264. 10.1016/j.peptides.2005.01.025 16274853 10.1016/j.peptides.2005.01.025 PMC1679957

[59] Lee M, Kim A, Conwell IM et al (2008) Effects of selective modulation of the central melanocortin-3-receptor on food intake and hypothalamic POMC expression. Peptides 29:440–447. 10.1016/j.peptides.2007.11.005 18155809 10.1016/j.peptides.2007.11.005 PMC2278043

[60] Montgomery MK, Hallahan NL, Brown SH et al (2013) Mouse strain-dependent variation in obesity and glucose homeostasis in response to high-fat feeding. Diabetologia 56:1129–1139. 10.1007/s00125-013-2846-8 23423668 10.1007/s00125-013-2846-8

[61] Chua S, Li Y, Liu SM et al (2010) A susceptibility gene for kidney disease in an obese mouse model of type II diabetes maps to chromosome 8. Kidney Int 78:453–462. 10.1038/ki.2010.160 20520596 10.1038/ki.2010.160 PMC3998677

[62] Krashes MJ, Lowell BB, Garfield AS (2016) Melanocortin-4 receptor-regulated energy homeostasis. Nat Neurosci 19:206–219. 10.1038/nn.4202 26814590 10.1038/nn.4202 PMC5244821

[63] Krude H, Biebermann H, Luck W, Horn R, Brabant G, Gruters A (1998) Severe early-onset obesity, adrenal insufficiency and red hair pigmentation caused by POMC mutations in humans. Nat Genet 19:155–157. 10.1038/509 9620771 10.1038/509

[64] Lee M, Poh LK, Kek BL, Loke KY (2008) Novel melanocortin 4 receptor mutations in severely obese children. Clin Endocrinol 68:529–535. 10.1111/j.1365-2265.2007.03071.x 10.1111/j.1365-2265.2007.03071.x 17941900

[65] Yu H, Chhabra KH, Thompson Z et al (2020) Hypothalamic POMC deficiency increases circulating adiponectin despite obesity. Mol Metab 35:100957. 10.1016/j.molmet.2020.01.021 32244188 10.1016/j.molmet.2020.01.021 PMC7082555

[66] Timper K, Brüning JC (2017) Hypothalamic circuits regulating appetite and energy homeostasis: pathways to obesity. Dis Model Mech 10:679–689. 10.1242/dmm.026609 28592656 10.1242/dmm.026609 PMC5483000

[67] Ghamari-Langroudi M, Cakir I, Lippert RN et al (2018) Regulation of energy rheostasis by the melanocortin-3 receptor. Sci Adv 4:eaat0866. 10.1126/sciadv.aat0866 30140740 10.1126/sciadv.aat0866 PMC6105298

[68] Di Micioni E, Botticelli L, Tomassoni D, Tayebati SK, Di Micioni MV, Cifani C (2020) The Melanocortin System behind the dysfunctional eating behaviors. Nutrients 12:3502. 10.3390/nu12113502 33202557 10.3390/nu12113502 PMC7696960

[69] Nogueiras R, Wiedmer P, Perez-Tilve D et al (2007) The central melanocortin system directly controls peripheral lipid metabolism. J Clin Invest 117:3475–3488. 10.1172/JCI31743 17885689 10.1172/JCI31743 PMC1978426

[70] Morgan DA, McDaniel LN, Yin T et al (2015) Regulation of glucose tolerance and sympathetic activity by MC4R signaling in the lateral hypothalamus. Diabetes 64:1976–1987. 10.2337/db14-1257 25605803 10.2337/db14-1257 PMC4439564

[71] Braun TP, Marks DL (2011) Hypothalamic regulation of muscle metabolism. Curr Opin Clin Nutr Metab Care 14:237–242. 10.1097/MCO.0b013e328345bbcd 21502918 10.1097/MCO.0b013e328345bbcd

[72] Lin EE, Scott-Solomon E, Kuruvilla R (2021) Peripheral innervation in the regulation of glucose homeostasis. Trends Neurosci 44:189–202. 10.1016/j.tins.2020.10.015 33229051 10.1016/j.tins.2020.10.015 PMC7904596

[73] Franckhauser S, Munoz S, Elias I, Ferre T, Bosch F (2006) Adipose overexpression of phosphoenolpyruvate carboxykinase leads to high susceptibility to diet-induced insulin resistance and obesity. Diabetes 55:273–280. 10.2337/diabetes.55.02.06.db05-0482 16443757 10.2337/diabetes.55.02.06.db05-0482

[74] Frasson D, Boschini RP, Chaves VE et al (2012) The sympathetic nervous system regulates the three glycerol-3P generation pathways in white adipose tissue of fasted, diabetic and high-protein diet-fed rats. Metabolism 61:1473–1485. 10.1016/j.metabol.2012.03.014 22592131 10.1016/j.metabol.2012.03.014

[75] Klein S, Gastaldelli A, Yki-Järvinen H, Scherer PE (2022) Why does obesity cause diabetes? Cell Metabol 34:11–20. 10.1016/j.cmet.2021.12.012 10.1016/j.cmet.2021.12.012 PMC8740746 34986330

[76] Stern JH, Rutkowski JM, Scherer PE (2016) Adiponectin, Leptin, and fatty acids in the Maintenance of Metabolic Homeostasis through adipose tissue crosstalk. Cell Metab 23:770–784. 10.1016/j.cmet.2016.04.011 27166942 10.1016/j.cmet.2016.04.011 PMC4864949

[77] Zurlo F, Larson K, Bogardus C, Ravussin E (1990) Skeletal muscle metabolism is a major determinant of resting energy expenditure. J Clin Invest 85:1423–1427. 10.1172/JCI114857 10.1172/JCI114857 PMC296885 2243122

[78] Mengeste AM, Rustan AC, Lund J (2021) Skeletal muscle energy metabolism in obesity. Obesity 29:1582–1595. 10.1002/oby.23227 34464025 10.1002/oby.23227

[79] Yagi S, Kadota M, Aihara KI et al (2014) Association of lower limb muscle mass and energy expenditure with visceral fat mass in healthy men. Diabetol Metab Syndr 26:27. 10.1186/1758-5996-6-27 10.1186/1758-5996-6-27 PMC3945716 24571923

[80] Benarroch E (2024) What is the role of the sympathetic system in skeletal muscle? Neurology 102:e209488. 10.1212/wnl.0000000000209488 38710007 10.1212/wnl.0000000000209488

[81] Delbono O, Rodrigues ACZ, Bonilla HJ, Messi ML (2021) The emerging role of the sympathetic nervous system in skeletal muscle motor innervation and sarcopenia. Ageing Res Rev 67:101305. 10.1016/j.arr.2021.101305 33610815 10.1016/j.arr.2021.101305 PMC8049122

[82] Straka T, Vita V, Prokshi K et al (2018) Postnatal Development and Distribution of Sympathetic Innervation in mouse skeletal muscle. Int J Mol Sci 1935. 10.3390/ijms19071935 10.3390/ijms19071935 PMC6073285 29966393

[83] Wang ZM, Messi ML, Rodrigues ACZ, Delbono O (2022) Skeletal muscle sympathetic denervation disrupts the neuromuscular junction postterminal organization: a single-cell quantitative approach. Mol Cell Neurosci 120:103730. 10.1016/j.mcn.2022.103730 35489637 10.1016/j.mcn.2022.103730 PMC9793435

[84] Vanhaesebrouck AE, Beeson D (2019) The congenital myasthenic syndromes: expanding genetic and phenotypic spectrums and refining treatment strategies. Curr Opin Neurol 32:696–703. 10.1097/WCO.0000000000000736 31361628 10.1097/WCO.0000000000000736 PMC6735524

[85] McMacken GM, Spendiff S, Whittaker RG et al (2019) Salbutamol modifies the neuromuscular junction in a mouse model of ColQ myasthenic syndrome. Hum Mol Genet 28:2339–2351. 10.1093/hmg/ddz059 31220253 10.1093/hmg/ddz059 PMC6606850

[86] Wang ZM, Messi ML, Grinevich V, Budygin E, Delbono O (2020) Postganglionic sympathetic neurons, but not locus coeruleus optostimulation, activates neuromuscular transmission in the adult mouse in vivo. Mol Cell Neurosci 109:103563. 10.1016/j.mcn.2020.103563 33039519 10.1016/j.mcn.2020.103563 PMC8049128

[87] Joassard OR, Bélanger G, Karmouch J et al (2015) HuR mediates changes in the Stability of AChR β-Subunit mRNAs after skeletal muscle denervation. J Neurosci 35:10949–10962. 10.1523/JNEUROSCI.1043-15.2015 26245959 10.1523/JNEUROSCI.1043-15.2015 PMC6605275

[88] Nishino T, Ranade SS, Pelonero A et al (2023) Single-cell multimodal analyses reveal epigenomic and transcriptomic basis for birth defects in maternal diabetes. Nat Cardiovasc Res 2:1190–1203. 10.1038/s44161-023-00367-y 10.1038/s44161-023-00367-y PMC11343316 39183978

