# Supplementary material for: Alx3 deficiency disrupts energy homeostasis, alters body composition, and impairs hypothalamic regulation of food intake
Source: Cell Mol Life Sci. 2024 Aug 12;81(1):343. doi: 10.1007/s00018-024-05384-z (PMC11335267; doi:10.1007/s00018-024-05384-z)
Supplement: Supplementary file 1 — Supplementary Material 1 [file 18_2024_5384_MOESM1_ESM.pdf]

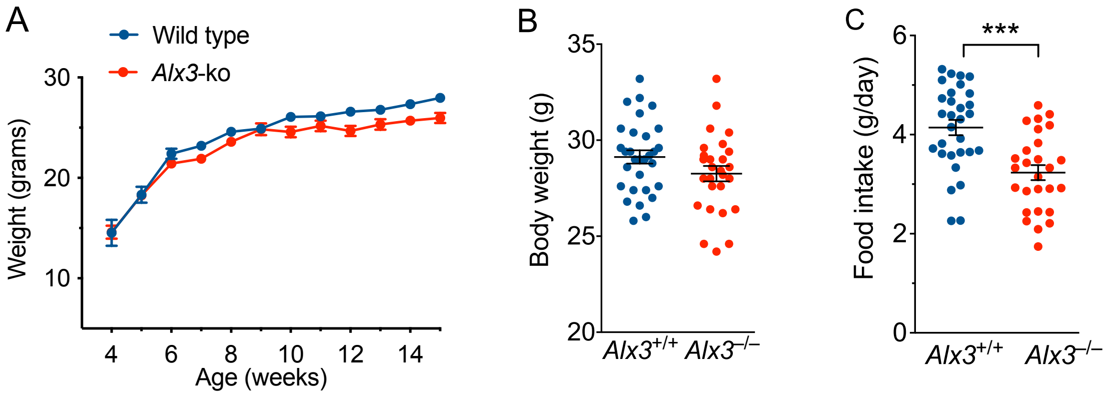

Supplementary Figure 1. Body weight and food intake. A) Evolution of body weight of *Alx3*-deficient and control wild-type mice monitored between 4 and 15 weeks of age. Data represent the mean ± SEM of 14 animals in each group. B) Body weight determined at an age of 20 weeks. C) Daily food intake determined at an age of 20 weeks. Values represent the mean of the total amount of grams per day taken by each mouse during a four-day period. ****p*<0.001, Student's t-test (n = 30 wild type and 27 *Alx3*-deficient mice).

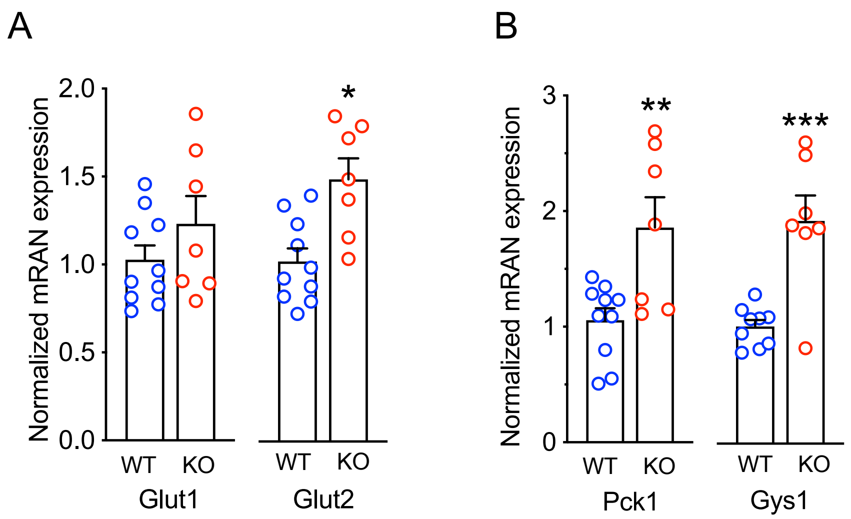

Supplementary Figure 2. Hepatic gene expression in HFD-fed mice. Expression of liver mRNA encoding glucose transporters (A) or glycogenic enzymes (B) in wild type (WT) od Alx3-deficient (KO) mice subjected to feeding with a HFD (n = 10 wild type and 7 Alx3-deficient mice per group. *p<0.05, **p<0.01 and ***p<0.001, Student's t-test.

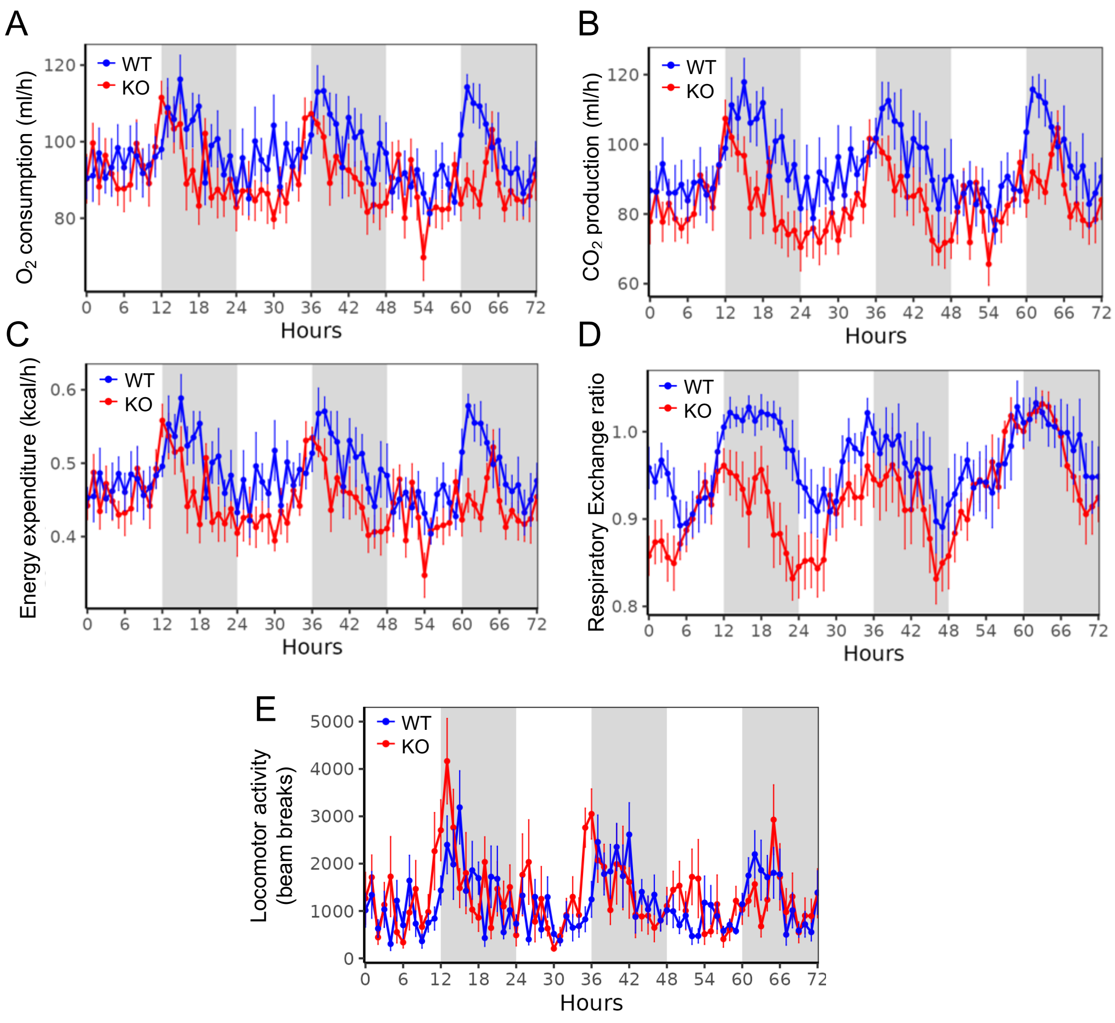

Supplementary Figure 3. Metabolic phenotype obtained by indirect calorimetry experiments. Graphs represent extended data from mice fed with standard chow diets obtained during the entire length of the experiments (72 hours), corresponding to those depicted in condensed form in Figure 3A-E. The dark phase of each 24-hour cycle is depicted in gray.

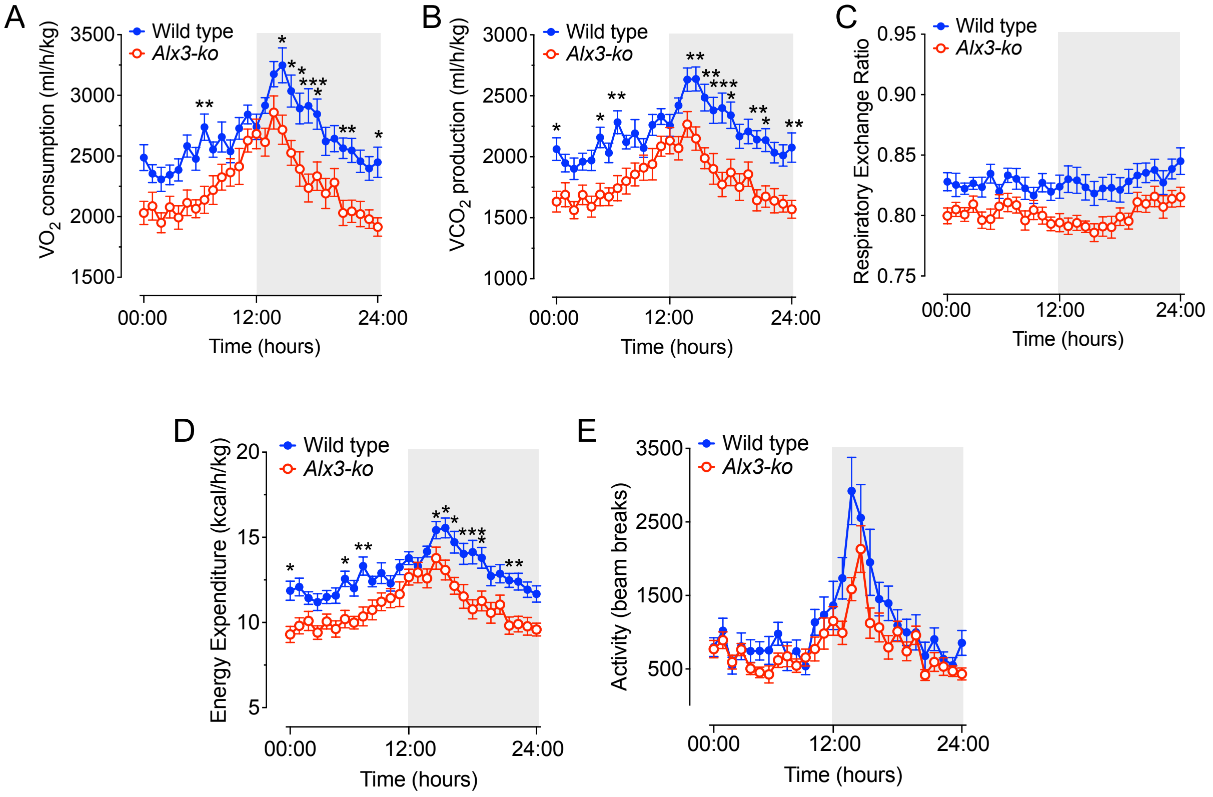

Supplementary Figure 4. Decreased respiratory exchange ratio and energy expenditure in *Alx3*-deficient mice fed with a high fat diet. Indirect calorimetry measurements showing O_2 consumption (A), CO_2 production (B), RER (C), energy expenditure (D) and locomotor activity (E). The data represent the mean values obtained at each time point over a period of three days for each mouse. Extended data showing the values corresponding to the entire three-day period are shown in Supplementary Figure 5. The dark phase of the cycle is represented in gray. Data represent mean ± SEM. **p*<0.05, ***p*<0.01, ****p*<0.001, two-way ANOVA followed by Bonferroni transformation (n = 14 wild type and 15 *Alx3*-ko mice).

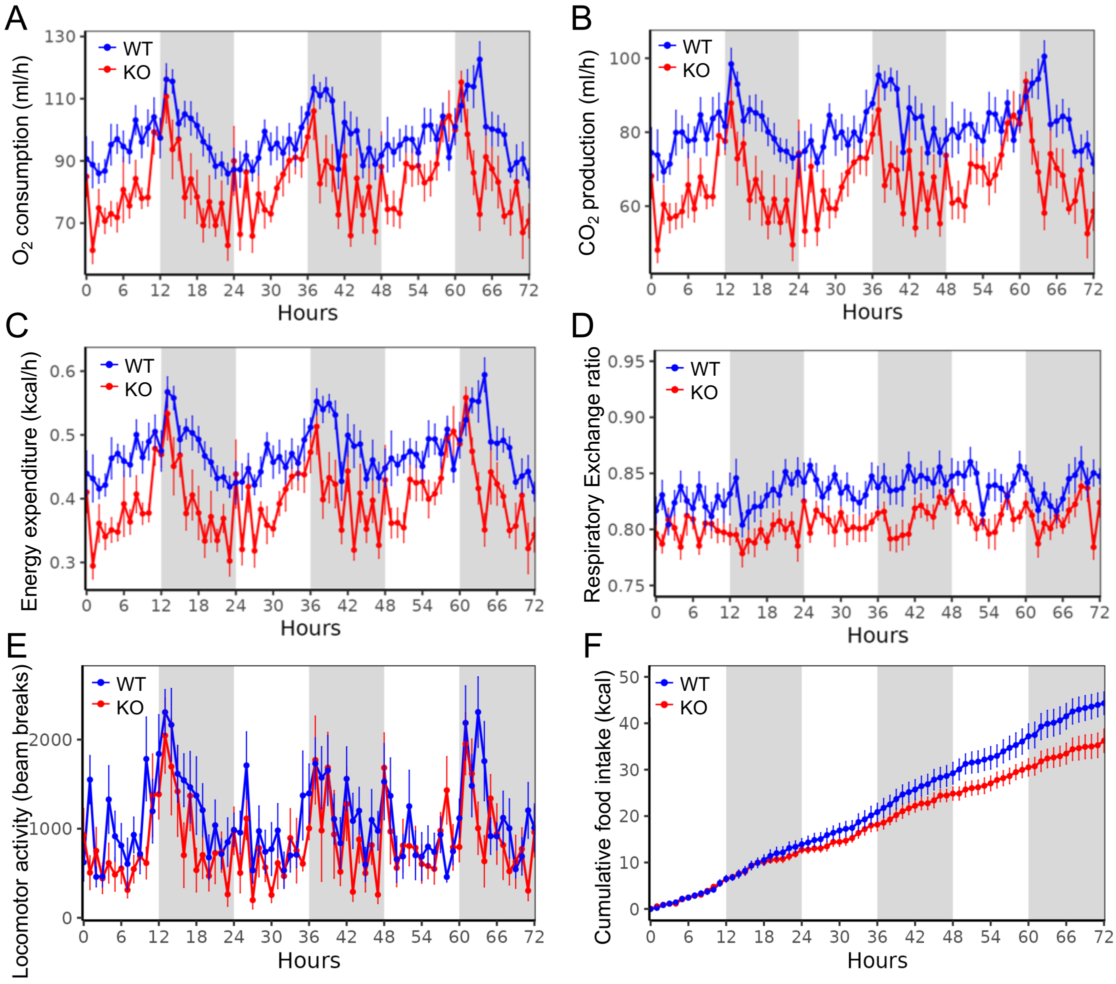

Supplementary Figure 5. Metabolic phenotype obtained by indirect calorimetry experiments. A-E) Graphs represent extended data from mice fed with high fat diet obtained during the entire length of the experiments (72 hours), corresponding to those depicted in condensed form in Supplementary Figure 4A-E. F) Cumulative quantification of the amount of food eaten during three days. The dark phase of each 24-hour cycle is depicted in gray.

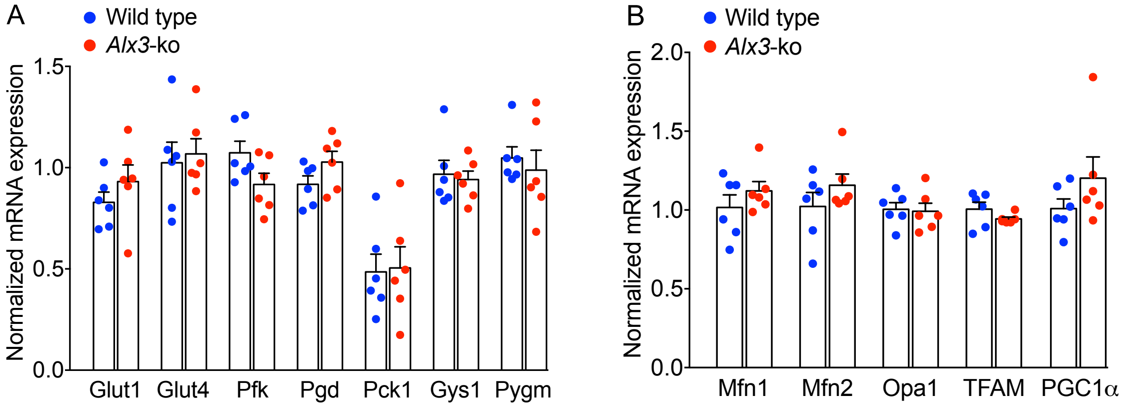

Supplementary Figure 6. Expression of metabolic and mitochondrial markers in muscle. Levels of mRNAs encoding proteins involved in glucose transport and metabolism (A) or mitochondrial function (B) in the gastrocnemius muscle of wild type or *Alx3*-deficient mice (n=6 per genotype). Glut1 and Glut4, glucose transporters 1 and 4; Pfk, phosphofructokinase; Pgd, phosphogluconate dehydrogenase; Pck1, phosphoenolpyruvate carboxykinase 1; Gys1, glycogen synthase 1; Pygm, Glycogen phosphorylase; Mfn1 and Mfn2, mitofusin 1 and 2; Opa1, mitochondrial dynamin-like GTPase; TFAM, mitochondrial transcription factor A. PGC1α, peroxisome proliferator-activated receptor γ co-activator 1α.

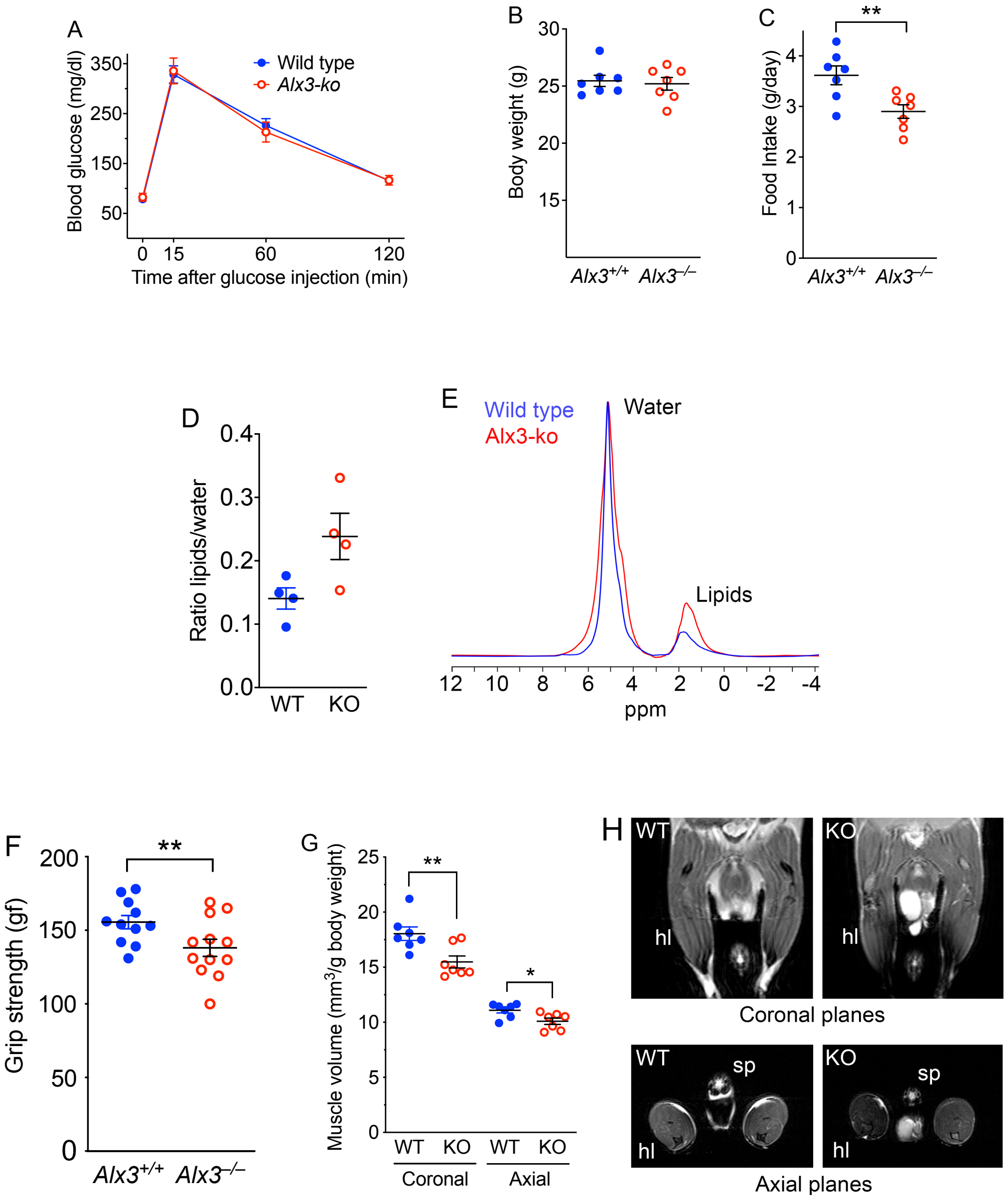

Supplementary Figure 7. Phenotype of *Alx3*-deficient mice in a pure C57BL/6J background. A) Glucose tolerance tests performed in wild-type (n = 10) or *Alx3*-deficient (n = 11) mice that received an intraperitoneal injection of glucose (2 g/kg body weight) after an overnight fast. B and C) Body weight and food intake determined in 20-week old mice. The food intake values represent the mean of the total amount of grams per day taken by each mouse during a four-day period (n=7 mice per genotype). D) Quantification of the total proportion of body lipids by ^{1}H-MRS (n=4 per genotype). E) Representative spectra from which measurements were calculated. F) Forelimbs muscle strength as assessed by the grip test (n=11 wild type and 12 *Alx3*-deficient mice). G) Muscle volume relative to body weight calculated from coronal or axial images generated by MRI from hind limbs of wild type or *Alx3*-ko mice. H) Representative MRI coronal or axial planes used for quantification. hl, hind limb; sp, spine. **p*<0.05, ***p*<0.01, Student's t-test.

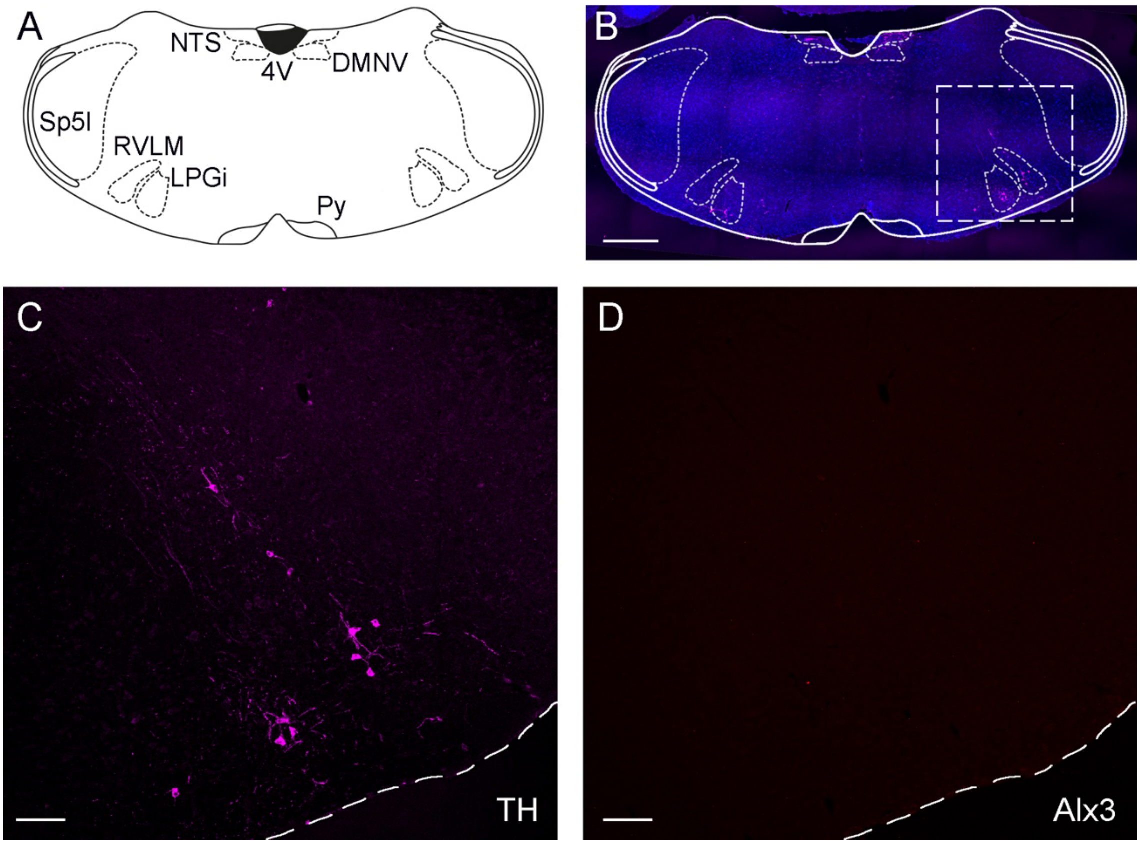

Supplementary Figure 8. Absence of Alx3 in sympathetic nuclei of the medulla oblongata. A) Schematic depiction of a coronal section of the mouse brainstem. 4V, fourth ventricle; DMNV, dorsal motor nucleus of the vagus nerve; LPGi, lateral paragigantocellular nucleus; NTS, nucleus tractus solitarius; Py, pyramidal tract; RLVM, rostral ventrolateral medulla; Sp5I, spinal trigeminal nucleus interpolar. B) High resolution confocal microscopy image of a representative coronal section of a mouse brainstem showing the presence of tyrosine hydroxylase-positive neurons and the absence of Alx3 immunostaining. Cell nuclei were labeled with DAPI, and Alexa-Fluor 647 and 546 were used for tyrosine hydroxylase and Alx3 immunofluorescence, respectively. The location of the main structures indicated in A is shown superimposed. The area shown at higher magnification in C and D is indicated with a square. C) High magnification of the ventrolateral medulla showing tyrosine hydroxylase positive neurons in the region corresponding to the RVLM and LPGi identified with the far-red fluorescent channel. D) The same region observed with the red channel showing the absence of Alx3-dependent immunofluorescence. Scale bar: 500 μm in B and 100 μm in C and D.

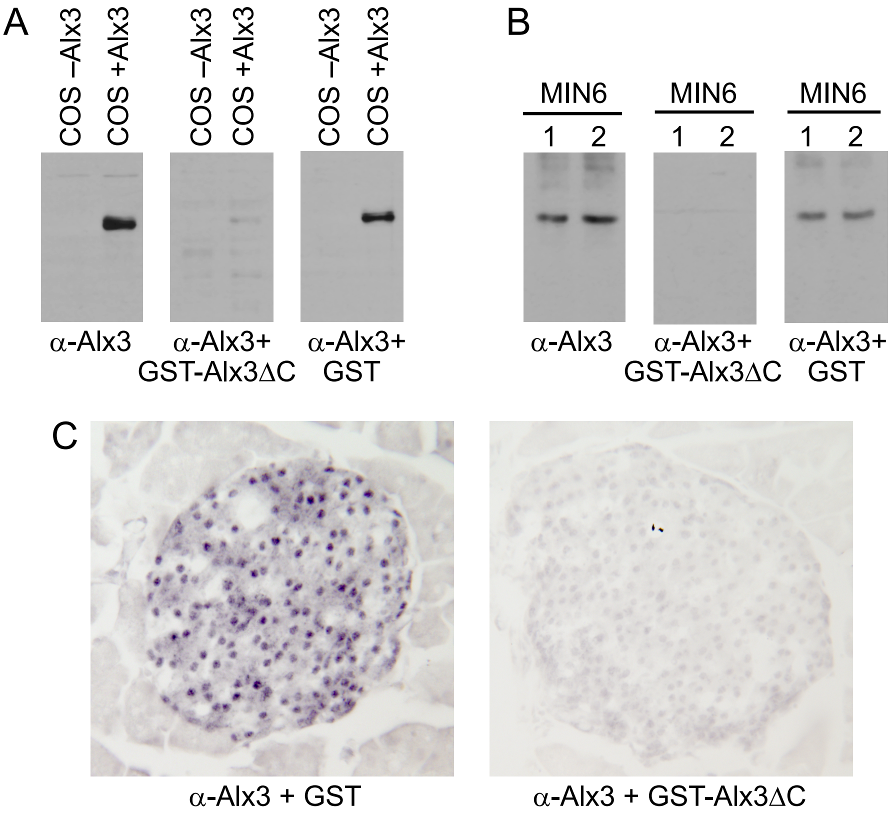

Supplementary Figure 9. Specificity control for the Alx3 antibody (α-Alx3). A and B) Western blots carried out with lysates from COS cells transfected with an empty vector (COS –Alx3) or with an Alx3 expression plasmid (COS +Alx3) (A), or from MIN6 pancreatic β cells (B). The numbers on top of each lane indicate individual plates from which lysates were prepared. Where indicated, the antibody had been pre-absorbed with GST-Alx3ΔC fusion protein or with control GST. C) Representative mouse pancreatic islets showing Alx3-specific DAB immunoperoxidase staining when the antibody had been pre-absorbed with control GST (left panel) but not when the pre-absorption was carried out with GST-Alx3ΔC (right panel). In both cases, note the absence of Alx3-immunoreactive cells in the exocrine pancreas surrounding the islets, as expected.

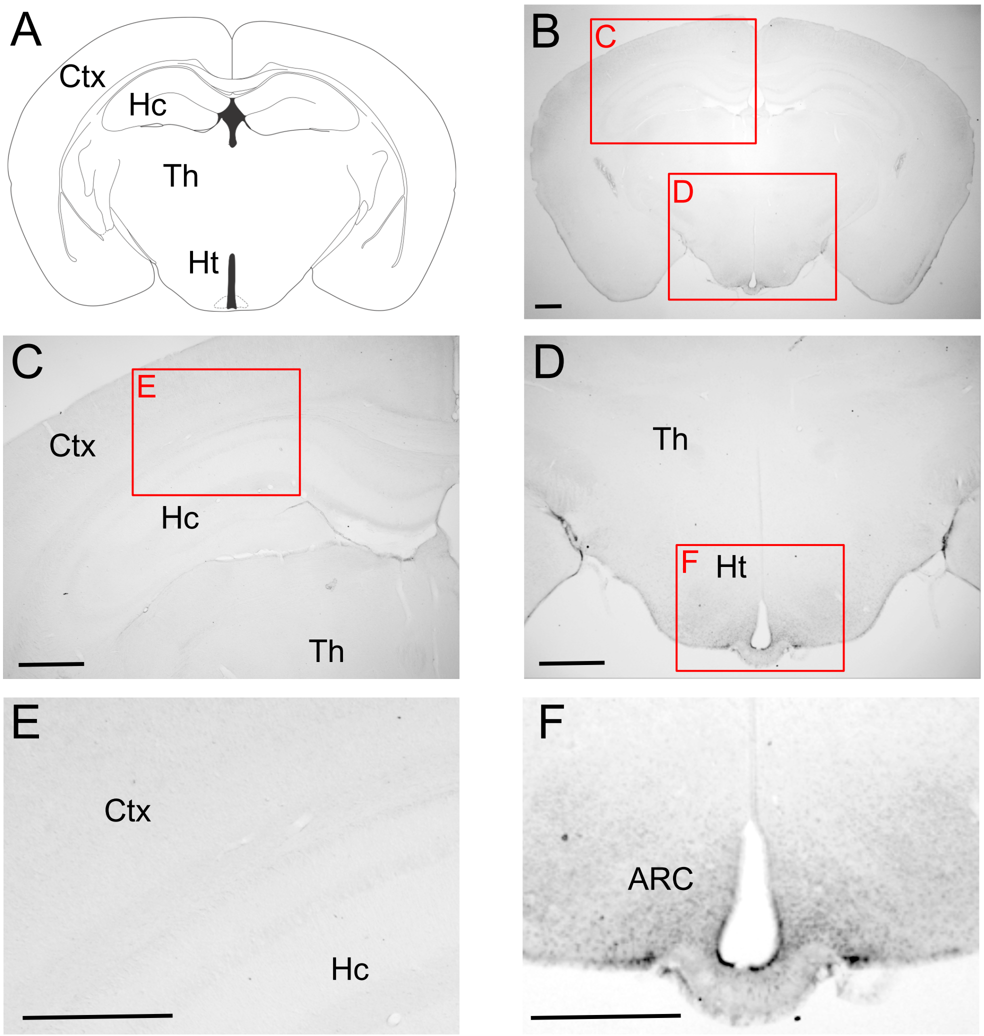

Supplementary Figure 10. Restricted expression of Alx3 to the hypothalamus. A) Schematic depiction of a coronal section of the mouse brain at a level of -1.82 mm from Bregma. The drawing was modified from Paxinos, G. and Franklin, K.B.J., The mouse brain in stereotaxic coordinates, Academic Press, San Diego, 2001. B) Coronal section of a wild type mouse brain processed for immunohistochemistry with the R2Ib2 Alx3 antibody. Note the almost complete absence of immunoreactivity except for a small region in the ventral hypothalamus. Red rectangles indicate the brain regions depicted in panels C and D at higher magnification. In turn, in panels C and D red rectangles indicate the brain regions depicted in panels E and F at higher magnification. Abbreviations: ARC, arcuate nucleus; Ctx, Cortex; Hc, Hippocampus; Ht, Hypothalamus; Th, Thalamus. Scale bars, 500 µm.

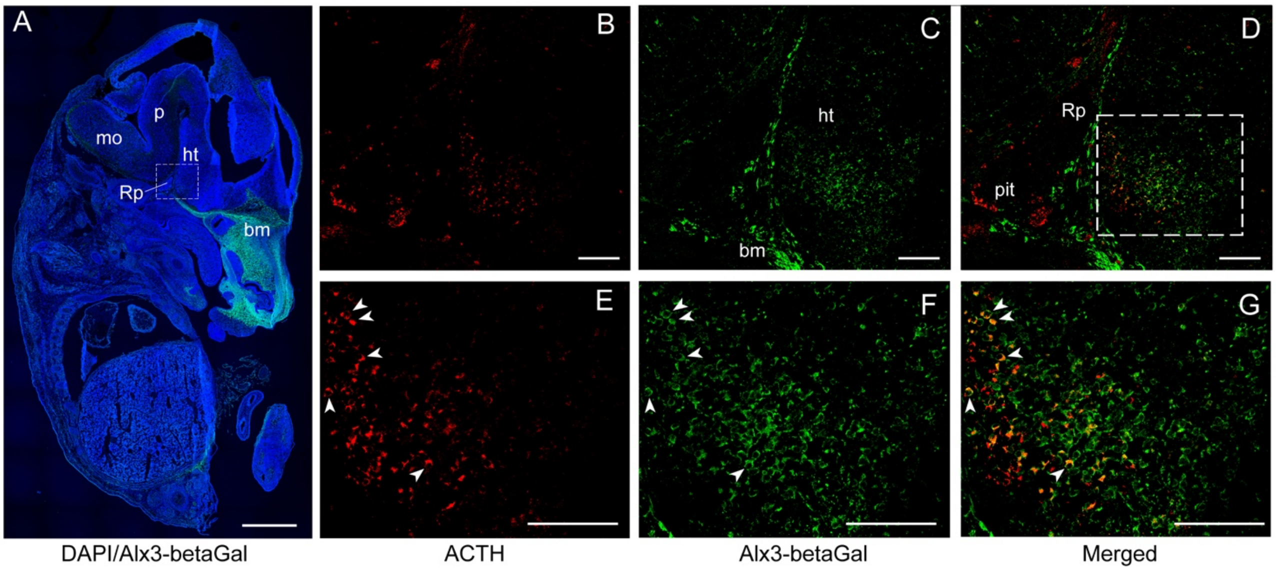

Supplementary Figure 11. Expression of Alx3 in the hypothalamic primordium during embryonic development. A) Confocal microscopy image of a representative sagittal section of an E13.5 Alx3-deficient mouse embryo expressing Alx3-β-galactosidase. The green color represents immunofluorescence detected with an anti β-galactosidase antibody. The blue color corresponds to cell nuclei labeled with DAPI. The rectangle indicates the area shown in panels B-G at higher magnification. B-G) Confocal microscopy images corresponding to the region containing the developing hypothalamus and pituitary labeled with antibodies against ACTH (red) and β-galactosidase (green). The rectangle in panel D corresponds to images shown at higher magnification in panels E-G. Examples of cells co-expressing ACTH and Alx3-β-galactosidase are indicated by arrowheads. Abbreviations: bm, bone mesenchyme of the basal sphenoid and rostronasal regions; ht, hypothalamus; mo, medulla oblongata; p, pons; pit, pituitary; Rp, residual lumen of Rathke's pouch. Scale bar, 1 mm (A) or 100 μm (B-G).

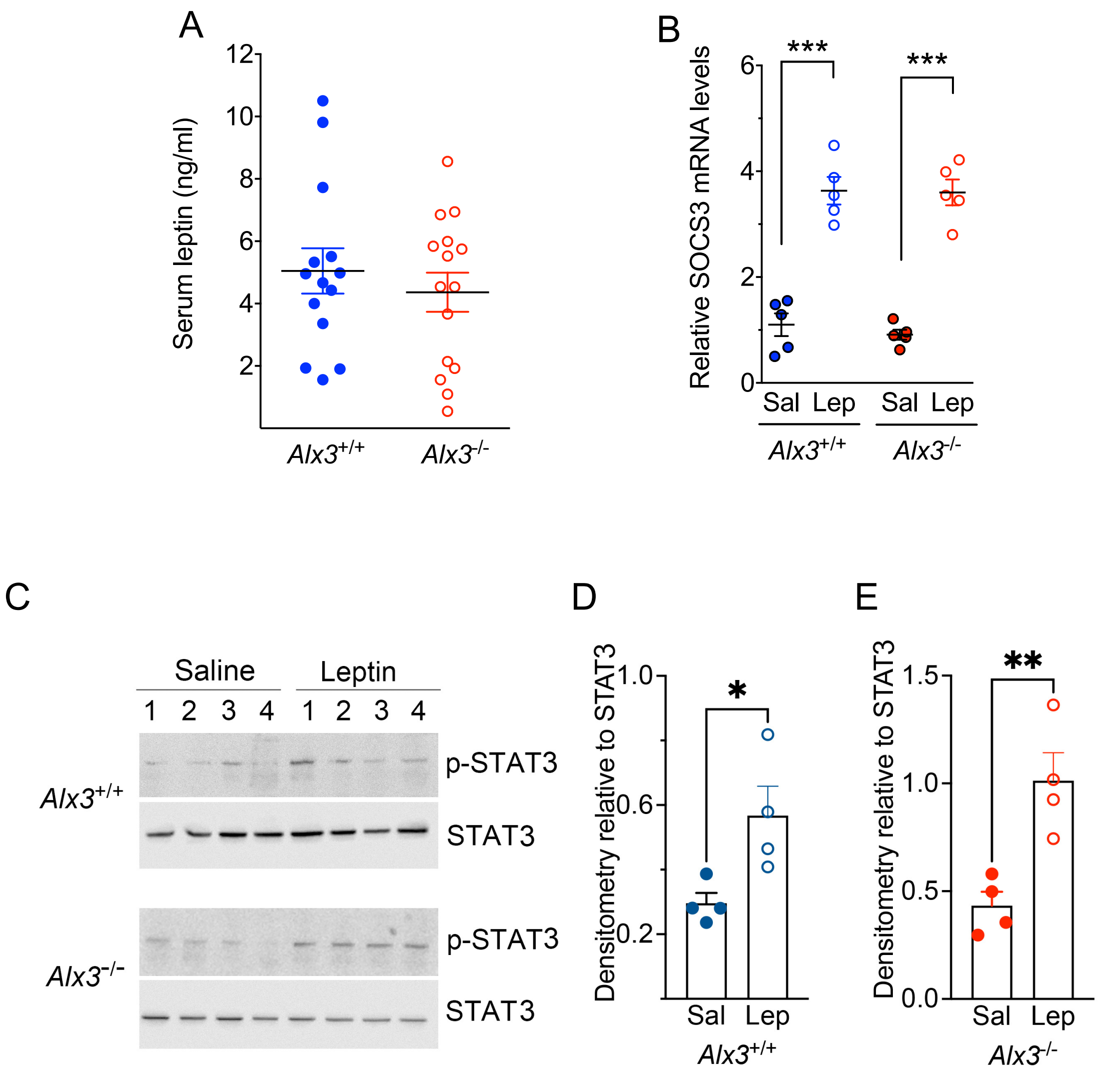

Supplementary Figure 12. Normal leptin function in *Alx3*-deficient mice. A) Serum leptin levels after an overnight fast (Alx3$^{+/+}$, n = 14; Alx3$^{-/-}$, n = 15). B) Expression of SOCS3 mRNA in the arcuate nucleus of mice following an intraperitoneal injection of saline (Sal) of leptin (Lep) (n = 5 per genotype). C) Western blot showing phosphorylation of STAT3 in arcuate nucleus protein extracts following intraperitoneal administration of leptin. The numbers on top of each lane indicate individual mice from which samples were obtained. D and E) Densitometric quantifications of the intensities of the bands corresponding to each genotype. **p*<0.05, ***p*<0.01, ****p*<0.001, Student's t-test.

Supplementary Table 1. Oligonucleotides and TaqMan probes used in RT-qPCR experiments.

AChE	CGGAGGCTCTCATCAATACTGG	GGGACCCCGTAAACCAGAAAG
AChRa	CTCTCGACTGTTCTCCTGCTG	GTAGACCCACGGTGACTTGTA
AchRb	CATCATCGCTCACCCCAC	ACGGTCCACAACCATGGC
AdRA1	CATTGGGCCACAGACCTACT	CAAGGGAGAGAATCCAGCAG
AgRP	TaqMan Probe Mm00475829_g1 (Applied Biosystems)	
Alx3	TaqMan Probe Mm01204737_m1 (Applied Biosystems)	
Cart	TaqMan Probe Mm04210469_m1 (Applied Biosystems)	
ChAT-ps1	CCATTGTGAAGCGGTTTGGG	GCCAGGCGGTTGTTTAGATACA
ChAT-ps2	GACCAGCTAAGGTTTGCAGC	CAGGAAGCCGGTATGATGAGA
EpoR	ACGCTTGGAAGACTTGGTGTG	TGTTGGCAGTGAACACCAGAA
Esrrg	AAGATCGACACATTGATTCCAGC	CATGGTTGAACTGTAACTCCCAC
Fasn	TCTGGGCCAACCTCATTGGT	GAAGCTGGGGGTCCATTGTG
Gdnf	TCCAACTGGGGGTCTACGG	GCCACGACATCCCATAACTTCAT
Gfar1	CACTCCTGGATTTGCTGATGT	AGTGTGCGGTACTTGGTGC
GHSR	AAACAGACAGTGAAGATGCTTGCTG	GGCTCGAAAGACTTGGAAAACAG
GLP1R	GGGTCTCTGGCTACATAAGGACAAC	AAGGATGGCTGAAGCGATGAC
Glut1	CAGTTCGGCTATAACACTGGTG	GCCCCCGACAGAGAAGATG
Glut4	GTAACTTCATTGTCGGCATGG	AGCTGAGATCTGGTCAAACG
Gys1	GAACGCAGTGCTTTTCGAGG	CCAGATAGTAGTTGTCACCCCAT
Lept	GTGGCTTTGGTCCTATCTGTC	CGTGTGTGAAATGTCATTGATCC
LeptinR	ACACTGAAGGGAAGACACTG	GGGTTCTTAGGTAATGGCTCC
Magel2	TaqMan Probe Mm00844026_s1 (Applied Biosystems)	
MC3R	CTGTAGCAACGGGTGTCGG	ATCAGCCTGCCTCATCCC
MC4R	CAAGAACCTGCACTCACCCA	GACCCATTCGAAACGCTCAC
Mnf1	CCTACTGCTCCTTCTAACCCA	AGGGACGCCAATCCTGTGA
Mnf2	AGAACTGGACCCGGTTACCA	CACTTCGCTGATACCCCTGA
Mstn	CAGGAGAAGATGGGCTGAAT	GAGTGCTCATCGCAGTCAAG
Myog	TCACATAAGGCTAACACCCAG	GGAATTCGAGGCATATTATGA
NPY	TaqMan Probe Mm00445771_m1 (Applied Biosystems)	
Opa1	TGGAAAATGGTTCGAGAGTCAG	CATTCCGTCTCTAGGTTAAAGCG
Pax6	ATGCCCAGCTTCACCATG	GAACTGACACTCCAGGTG
Pck1	CTGCATAACGGTCTGGACTTC	CAGCAACTGCCCGTACTCC
Pfk	TGTGGTCCGAGTTGGTATCTT	GCACTTCCAATCACTGTGCC
PGC1a	AGCCGTGACCACTGACAACGAG	GCTGCA TGGTTCTGAGTGCT AAG
Pgd	ATGGCCCAAGCTGACATTG	GCACAGACCACAAATCCATGAT
Pomc	TaqMan Probe Mm00435874_m1 (Applied Biosystems)	
Pygm	CTTAGCCGGAGTGGAAAATGT	GTAATCTCTCGGAGTAGCCACA
Rbfox3	ATCGTAGAGGGACGGAAAATTGA	GTTCCCAGGCTTCTTATTGGTC

Smn1	TGCTCTAAAGAACGGTGACATTT	TTTCAAGGGAGTTGTGGCATT
SOCS3	ATGGTCACCCACAGCAAGTTT	TCCAGTAGAATCCGCTCTCCT
TFAM	GGAATGTGGAGCGTGCTAAAA	ACAAGACTGATAGACGAGGGG
Ucp1	ACTGCCACACCTCCAGTCATT	CTTTGCCTCACTCAGGATTGG

Supplementary Table 2. Antibodies used in western blots or histology.

Antibody	Source	Reference	Dilution
ACTH	NIDDK, National Institute of Health	AFP-156102789	1:200
β-Actin	Sigma-Aldrich	AC-15	1:10000
Alx3ΔC	M Vallejo laboratory	Biochem. J. 420: 199-208, 2013	1:2000
Choline acetyltransferase	Abcam	AB144P	1:500
β-Galactosidase	Abcam	Ab9361	1:1000
GAPDH	Abcam	ab8245	1:20000
cStat3 (79D7)	Cell Signaling Technology	#4904	1:1000
Phospho- Stat3 (Tyr705)	Cell Signaling Technology	9131	1:1000
Synaptobrevin 2	Synaptic Systems	104 202	1:500
Tyrosine hydroxylase (Immunofluorescence)	Merck	MAB5280	1:500
Tyrosine hydroxylase (western blot)	Chemicon International	ab152	1:1000
Vinculin	Santa Cruz	Sc-73614	1:5000
VMAT2	Merck	AB1598P	1:1000

Supplementary Table 3. ***CalR*** **analyses for data from mice fed with standard chow diet**

	**ANCOVA/Generalized linear model**					
	**Full day**		**Light**		**Dark**	
Effect	Body mass	Genotype	Body mass	Genotype	Body mass	Genotype
Energy expenditure	0.2142	0.0068**	0.5069	0.0192*	0.0818	0.0031**
O_2 consumption	0.1928	0.0124*	0.4538	0.0290*	0.0760	0.0065**
CO_2 production	0.3316	0.0011**	0.7368	0.0061**	0.1395	<0.001***

	**ANOVA**		
	**Full day**	**Light**	**Dark**
Effect	Group	Group	Group
Respiratory exchange ratio	0.0015**	0.0182*	0.0053**
Locomotor activity	0.4431	0.4558	0.4490

*p<0.05, **p<0.01 and ***p<0.001

Supplementary Table 4. ***CalR*** **analyses for data from mice fed with high fat diet**

	**ANCOVA/Generalized linear model**					
	**Full day**		**Light**		**Dark**	
Effect	Body mass	Genotype	Body mass	Genotype	Body mass	Genotype
Energy expenditure	<0.0029**	<0.001***	<0.001***	<0.001***	0.0151*	<0.001***
O_2 consumption	<0.0031**	<0.001***	<0.001***	<0.001***	0.0171*	<0.001***
CO_2 production	<0.0024**	<0.001***	<0.0012**	<0.001***	0.0106*	<0.001***

	**ANOVA**		
	**Full day**	**Light**	**Dark**
Effect	Group	Group	Group
Respiratory exchange ratio	0.0146*	0.0145*	0.0182*
Locomotor activity	0.0057**	0.0095**	0.0104*

*p<0.05, **p<0.01 and ***p<0.001
